# Temporal changes in the size resolved fractions of bacterial aerosols in urban and semi-urban residences

**DOI:** 10.1038/s41598-024-70495-3

**Published:** 2024-08-30

**Authors:** N. Grydaki, I. Colbeck, C. Whitby

**Affiliations:** School of Life Sciences, University of Essex, Colchester, CO4 3SQ UK

**Keywords:** Bioaerosols, Particle size, Indoor environment, Microbiome, Microbiology, Environmental sciences

## Abstract

Despite the significant amount of time spent in the domestic environment, culture-independent size distribution data of bioaerosols are largely missing. This study investigated the temporal changes in size-resolved bacterial aerosols in urban and semi-urban residential settings. Overall, airborne bacterial taxa identified in both sites were dispersed across particles of various sizes. qPCR analysis showed that outdoors bacteria dominated particles > 8 μm, whilst indoor bacterial loadings were greater with 1–2 μm (winter) and 2–4 μm (summer) ranges. Indoor and outdoor aerosols harboured distinct bacterial communities due to the dominance of human-associated taxa (*Staphylococcus*, *Micrococcus*, *Corynebacterium*) in indoor air. The aerosol microbiome exhibited significant temporal variation, with *Actinobacteria*, *Gammaproteobacteria* and *Bacilli* predominant indoors, whereas *Actinobacteria*, *Alphaproteobacteria* and *Gammaproteobacteria* were the most abundant taxa outdoors. The variation between the two residences was mostly driven by particles < 2 μm, whereas differences between indoors and outdoors were mostly influenced by particles > 2 μm. Source-tracking analysis estimated that household surfaces accounted for the greatest source proportion of bacteria, surpassing that of outdoor air, which varied due to natural ventilation throughout the year. Our findings provide new insights into the factors governing the aerosol microbiome in residential environments which are crucial for exposure assessment.

## Introduction

Since people in developed nations spend around 80–90% of their time in the built environment, they will likely be exposed to air pollution primarily indoors^1,2^. Indoor environments, such as in educational^3^, clinical^4^ and transport settings^5^, represent unique environments of exposure, and as such concerns about indoor exposure have increased. However, a large proportion of time spent indoors is in residential settings. As a result, it is important to assess the microbial exposure associated with air quality in residential environments.

Indoor air particles comprise of a complex mixture of microorganisms (bioaerosols) originating from both indoor and outdoor sources^6,7^. Yet, few studies of indoor environments consider paired indoor and outdoor bioaerosol samples together^7,8–10^. Moreover, although longitudinal investigations of bioaerosols in outdoor air have been common (e.g.,^11,12^), little is known about the temporal changes of the microbial abundance and diversity of aerosols in indoor environments^13–15^.

Indoor bioaerosols may comprise 16–68% to PM_10_^16^. Although microbial abundance and diversity of total suspended particulates have been well studied, the limited information on the size distribution of bioaerosols is mainly derived by culture-based studies^17–19^. To date, only a few studies have evaluated the size-resolved diversity of bioaerosols in indoor^20–22^ and outdoor^23–26^ environments using high throughput sequencing (HTS) approaches. Aerodynamic diameter strongly influences the fate of biological particles and human exposure and therefore, size-resolved data are of fundamental value for developing insights regarding health effects of exposure to bioaerosols^27^.

In order to gain a better insight into the factors driving the indoor air microbiome in domestic environments, a sampling study was performed over a seasonal cycle within an urban and semi-urban residential setting in the United Kingdom. The overall goal of this study was to investigate the seasonal variation of bacterial aerosols in these urban and semi-urban residential environments across different particle size fractions based on molecular approaches.

## Methods

### Sampling

#### Sampling sites

Two residential flats (located ~ 80 km apart) within the urban district of Stratford, London (population: 342,430, ONS, 2017, UK), and the semi-urban district of Colchester, Essex (population: 186,635, ONS, 2017, UK), were used in this study. Both residences had similar characteristics (e.g. size, layout, ventilation and occupancy) (Table S1) and were situated in riverside residential blocks of similar age (Fig.S1). Throughout the sampling periods, the occupants maintained their normal routines. Sampling was performed during winter (February), spring (May) and summer (August) in 2016. A summary with all sampling dates and details is given (Tables S2**, **S3).

#### Air sampling

For indoor sampling, equipment was placed at a central position in the living room, at a height of 1.5 m, whilst outdoor sampling was performed either on an outside balcony (semi-urban), or through the bedroom window with the door to the room kept closed (urban) (Fig.S1). Whole day (12 h) simultaneous indoor and outdoor collection of the size-distributed aerosol samples was carried out between 09:00–21:00 for three days per site and season. Two seven-stage May impactors^28^, that collect particles onto standard microscope glass slides, were deployed. The May impactor has aerodynamic cut-off diameters of 16, 8, 4, 2, 1, 0.5, and 0.25 µm, for stages 1 to 7, respectively, at 20 L/min. For outdoor sampling at the urban study house, the impactor inlet was connected to a sterile sampling tube (27-mm inner diameter and 50-cm length) which was passed through the window.

Glass slides (ThermoFisher Scientific, UK) used as collection substrates for May impactor stages were washed in 10% bleach solution (0.05% sodium hypochlorite) and rinsed with ultrapure water (Milli-Q, Millipore) and 70% (v/v) ethanol. Following each day’s sampling, the May impactor was sterilised with 70% (v/v) ethanol and each stage was autoclaved, with impactor parts also sterilised from both sides under UV light for 20 min in a UV crosslinker to remove DNA contamination. Assembling of impactors and loading of sampling substrates was carried out in a safety cabinet using aseptic techniques. All openings were sealed to prevent contamination from ambient air. After sampling, slides from the May impactor were removed and swabbed immediately using sterile nylon flock swabs (#552C, Copan Diagnostics) moistened with sterile 1 × PBS (10 mM phosphate, 137 mM NaCl and 2.7 mM KCl). Once the swabbing was complete, swabs were returned to the dry transport tubes and stored at −20 °C.

#### Source tracking sampling

Samples from potential sources which may account for attracting biological material, as well as deposition and resuspension of particles, were collected indoors. A 100 cm^2^ (10 cm × 10 cm) area, determined by a template made from autoclaved paper, was swabbed from the following five common surfaces in each house: bookcase shelf (living room), bench (kitchen), wooden floor (entrance hall), chest of drawers (bedroom) and bathtub abutment (bathroom) (Fig.S1). One extra sample was collected from the carpeted bedroom floor in the semi-urban flat (urban flat had no carpet). Surfaces were sampled in triplicate using nylon flocked swabs moistened with sterile PBS buffer. In order to investigate whether the riverside location affects the microbial composition of the indoor air in the study apartments, 50 ml water samples were also collected from 0–0.5 m depth from the adjacent rivers (River Colne in Colchester and Bow Back Rivers in East London). Swab samples were collected per each house for all three seasons, while river samples were only collected during spring and summer.

#### Environmental parameters monitoring

Parameters of the microclimate (temperature and relative humidity) in the study houses, as well as carbon dioxide concentrations, were recorded continuously at one-minute intervals using a Rotronic CP11 indoor air quality meter, side-by-side with bioaerosol sampling. Outdoor meteorological parameters, including temperature, relative humidity, wind speed and precipitation accumulation, for the duration of the sampling periods, were retrieved from WU Personal Weather Station Network (www.wunderground.com). All environmental data for both sites are presented in Table S4 and Fig.S2.

### Sample processing

#### DNA extraction

The tips of all swab samples collected from the May impactor slides (stages) and surfaces were cut using sterile scissors, inserted into 2-ml zirconium/silica-bead filled (0.1-mm, 0.5 g) screw-cap tubes containing 500 μl of 2.5% (v/v) SDS (10 mM Tris–HCl pH 8, 25 mM Na_2_EDTA pH 8, 100 mM NaCl and molecular biology grade water) and cells were lysed by beat beating using a Precellys Evolution tissue homogeniser (Bertin Instruments, France) for 3 × 60 s at 7200 rpm. An equal volume of phenol:chloroform:isoamyl alcohol (25:24:1) pH 8 was added and the tubes were centrifuged at 11,337 × g for 5 min. The supernatants were placed in new 2-ml microcentrifuge tubes and subjected to another centrifugation at 11,337 × g for 5 min. DNA was precipitated by the addition of equal volume of 100% HPLC-grade isopropanol and 1.5 µl GlycoBlue (15 mg/ml, Invitrogen Ambion) and incubated for 100 min at room temperature, followed by centrifugation at 11,337 × g for 30 min. DNA was then washed with 80% (v/v) ice-cold ethanol, air-dried and resuspended in 35 μl of sterile PCR-grade water.

Genomic DNA from the 50 ml water samples collected from the rivers was extracted by centrifugation at 8,000 × g for 30 min. The pellet was resuspended in 1.5 ml of the supernatant, transferred in Eppendorf tubes and centrifuged at 11,337 × g for 10 min. The pellet was dissolved in 500 μl extraction buffer, placed in 2-ml bead-beating tubes and cells were lysed as described previously.

#### Bacterial 16S rRNA gene quantification

16S rRNA bacterial gene abundances were quantified using the universal primers Bakt_341F (CCTACGGGNGGCWGCAG) and Bakt_805R (GACTACHVGGGTATCTAATCC), described by Herlemman et al.^29^. All real-time PCR runs were performed in duplicate on a CFX96 Real-Time System/C1000 thermal cycler (BioRad, USA). A reaction mixture (total volume 10 μl) comprised 5 μl (1X) SensiFAST™ SYBR® No-ROX Kit (Bioline), 0.2 μl of each primer (final concentration 200 nM), 3.6 μl microbial DNA-free water (Qiagen) and 1 μl DNA. The thermal cycling protocol was as follows: 95 °C for 3 min, followed by 40 cycles of 5 s of denaturation at 95 °C and 30 s of annealing and extension at 60 °C. A melt-curve analysis was also performed for every qPCR assay with a temperature gradient of 0.5 °C from 65 to 95 °C to confirm primer specificity. Genomic DNA extracted from *Escherichia coli* K-12 was used to make a standard curve. The amplification efficiency was between 90.3 and 98.5%, with correlation coefficient > 0.98. No-template controls included in each run yielded no products or their Ct value was between 36 and 39. Data were acquired by CFX Manager Software (BioRad). Comparisons with the standard curve gave the estimated 16S rRNA gene copy number, on the basis of genome size (4.64 Mbp) and 16S rRNA gene copies (7) per *E. coli* genome, in each qPCR reaction.

#### Illumina MiSeq sequencing of 16S rRNA gene

Amplicon sequencing was performed using the primers Bakt_341F/Bakt_805R, targeting the V3 and V4 regions of the bacterial 16S rRNA gene^29^, with overhang adapter sequences for compatibility with Illumina indices, according to the manufacturer instructions (Illumina Library Preparation Workflow). For the size-resolved aerosols, DNA samples from each one of the three days per each site and season were pooled for each size fraction (i.e. 7 indoor and 7 outdoor size-resolved samples were analysed per each house and season). Aerosol samples and negative controls (1 field blank swab sample per site and 3 extraction controls), as well as source-tracking samples, were sequenced as paired-end reads (2 × 300 bp) on an Illumina MiSeq platform with v3-chemistry and 20% (v/v) PhiX**.** All sequences generated have been deposited in NCBI under accession number PRJNA1078868. Sequencing data were processed as described previously^5^ and taxonomy assignment to bacterial OTUs (97% similarity threshold) was performed using the RDP database^30^. Following quality control, removal of unassigned reads and sequences belonging to non-bacterial domains, subtraction of sequences resulting from the blanks/control samples, normalisation and abundance-based OTU filtering (*n* ≤ 5 counts), a total of 1,213,097 sequences were obtained, represented by 7840 OTUs, for the entire dataset.

### Data analysis

Particle size distribution data were plotted using normalised concentration (dN/dlogd_p_) that is independent of the size bin width. *dN* is the bacterial concentration (i.e. 16S rRNA genes per m^3^ of air) in the size range and *dlogd*_*p*_ is the difference in the log of the size channel width. Since, it is well documented that, large particles do not follow the air pathways^31^, a 40 μm cut-off diameter was assumed for the upper size bound of the May impactor. The indoor-to-outdoor (I/O) concentration ratio was also calculated, based on the qPCR-based estimates of 16S rRNA gene abundance, as a quantitative indicator of the impact of outdoor air on indoor air concentrations.

Statistical analysis was performed within R computing environment. To assess seasonal variation of bacterial 16S rRNA gene abundance, when normality (Shapiro–Wilk test) and equal variance (Levene’s test) assumptions were met, one-way analysis of variance (ANOVA) was conducted. Group means were compared using independent samples *t*-test or the Welch’s test. Spearman’s rank correlation analysis was also used to assess potential associations. Significance reported for any analysis was defined as *p* < 0.05.

The taxonomic distribution of OTUs across samples at the phylum, class and genus level was determined using the microbiome analysis package *phyloseq*^32^ and visualised with barplots constructed using *ggplot2*^33^ within R. Indicator species analysis to identify airborne bacterial genera that are characteristic for each site and season, as well as for particle size fractions < 2 μm and > 2 μm, in respective indoor and outdoor environments, was performed based on the Indicator Value (*IndVal*) index using the “multipatt” function with 9999 permutations within the R package *indicspecies*^34^.

Alpha diversity (OTU richness and Shannon’s index) and beta diversity (Bray–Curtis dissimilarity metric) were estimated and visualised using *phyloseq* within R. 95% confidence ellipses based on the multivariate normal distribution in Principal Coordinates Analyses (PCoA) 2D-plots were computed using “stat_ellipse” within *ggplot2* R package. Ellipses to group and annotate sets of points were generated using “geom_mark_ellipse”, which is based on the Khachiyan algorithm, within the R package *ggforce*^35^. To estimate the effect of influencing factors (site, environment, season, particle size) on the bacterial composition and test for significant differences among groups of samples, permutation-based multivariate analysis of variance (PERMANOVA)^36^ was applied using “adonis2” function within the R package *vegan*^37^. To test if groups differed in their dispersion effect (within-group variation), permutation-based analysis for homogeneity of multivariate dispersions (PERMDISP) was performed using “betadisper” function within *vegan*. The number of permutations for both tests was set to 9999. Boxplots of pairwise Bray–Curtis dissimilarity of aerosol bacterial composition based on both indoor and outdoor air samples within each season and per each site were generated using R package *metagMisc* (https://github.com/vmikk/metagMisc).

The proportional contribution of potential sources to the indoor air microbiome of the size-resolved aerosol samples (sinks) was estimated using the Fast Expectation–mAximization microbial Source Tracking (*FEAST*) algorithm, implemented in R^38^, with the default settings. For each site per each season, the microbiomes of the outdoor air size-resolved aerosols, the interior surfaces and the adjacent river water were considered as sources.

## Results

### Size-resolved 16S rRNA gene abundance of bacterial aerosols

Overall, there was no significant difference in total (non-size resolved) aerosol 16S rRNA gene abundance between the two sites, indoors and outdoors (Fig.S3), except for summer where indoor concentrations were significantly higher in the urban residence compared to the semi-urban (*t*-test, *p* < 0.05). For both houses, the highest bacterial load indoors was generally observed during spring, and outdoors during summer. The indoor concentrations were lower than outdoors (I/O ratios < 1) only in summer, with the difference being significant only for the urban site (*t*-test, *p* < 0.05).

**Figure 1:**
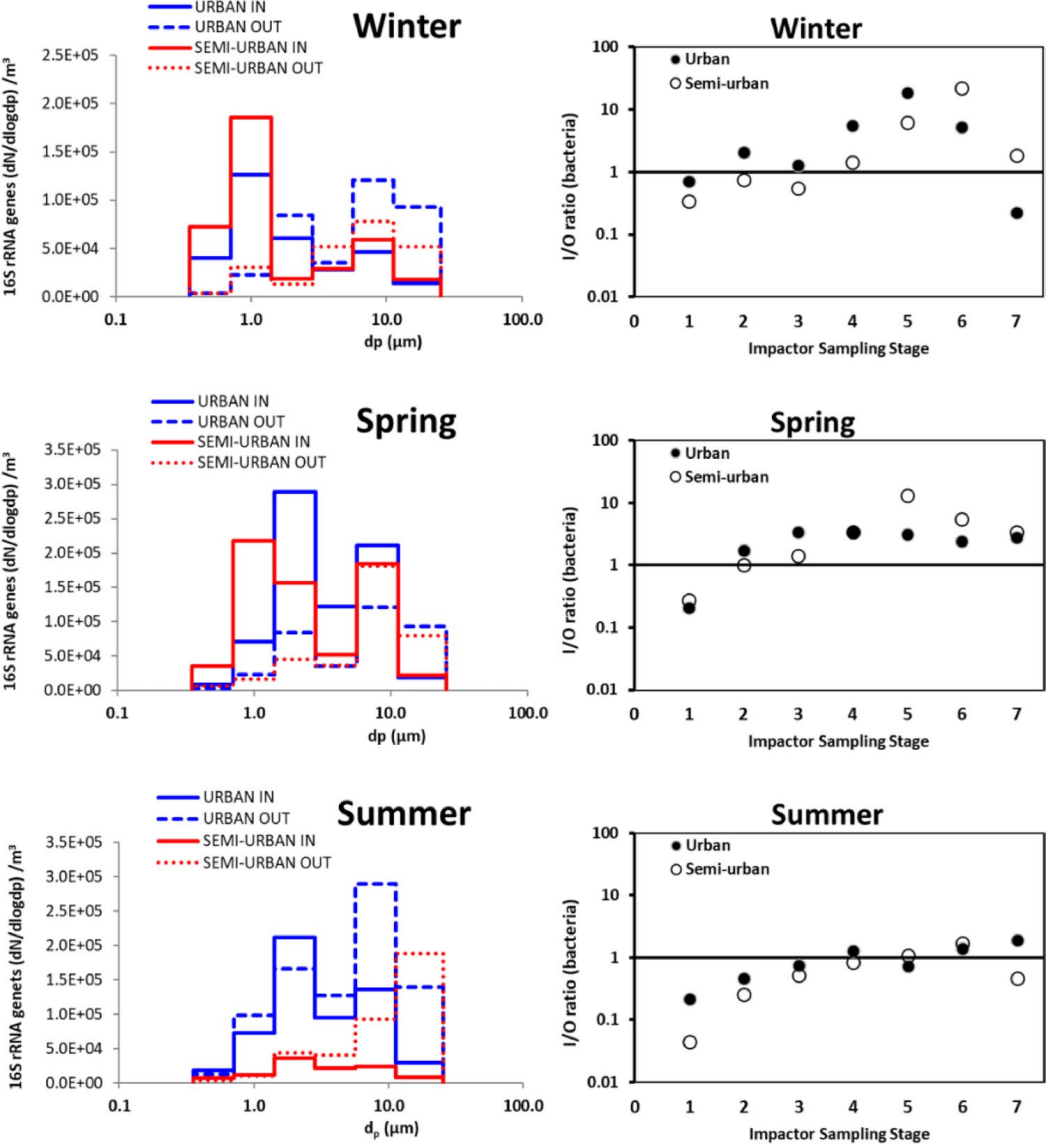
On the left panel: Seasonally averaged particle-size distributions (*n* = 3 days) of aerosol bacterial abundance determined by qPCR (y axis, dN/dlogdp 16S rRNA genes per m^3^ of air) indoors and outdoors for each site. An upper limit of particle size for the stage of > 16 μm is set at 40 μm. On the right panel: Size-resolved Indoor-to-Outdoor concentration ratios (I/O) for bacterial 16S rRNA genes per m^3^ of air in the urban (closed circles) and the semi-urban (open circles) residences across seasons. Each I/O ratio presented per season is an average over three days. Axis x represents the May impactor size bins 1 to 7, corresponding to aerodynamic cut-offs of 16, 8, 4, 2, 1, 0.5 and 0.25 μm, respectively.

The seasonally averaged particle-size distribution of bacterial gene copies demonstrated similar temporal trends for both sites (Fig. 1**)**. Outdoor bacterial aerosols were dominated by particles > 1 μm in aerodynamic diameter, across all seasons, with the highest concentrations for particles > 8 μm in most cases. In contrast, indoor profiles showed higher levels in the 1–2 μm size range in winter and 2–4 μm in summer at both sites. A mixed pattern was observed during spring, with higher abundances found in the 1–2 μm for the semi-urban and 2–4 μm for the urban houses. The only particles that consistently showed indoor-to-outdoor (I/O) ratios lower than unity for both residences were the ones with sizes greater than 16 µm (stage 1). Apart from the specific fraction, during winter and spring most stages demonstrated I/O ratios greater than unity, with the particles within the range 8–16 µm (stage 2) being close to unity. A clear decrease of the I/O ratio was observed during summer for particles of all sizes, with only the ratio for particles within the size range 0.5–1 µm (stage 6) remaining greater than unity for both sites.

### Size-resolved taxonomic composition of bacterial aerosols

**Figure 2:**
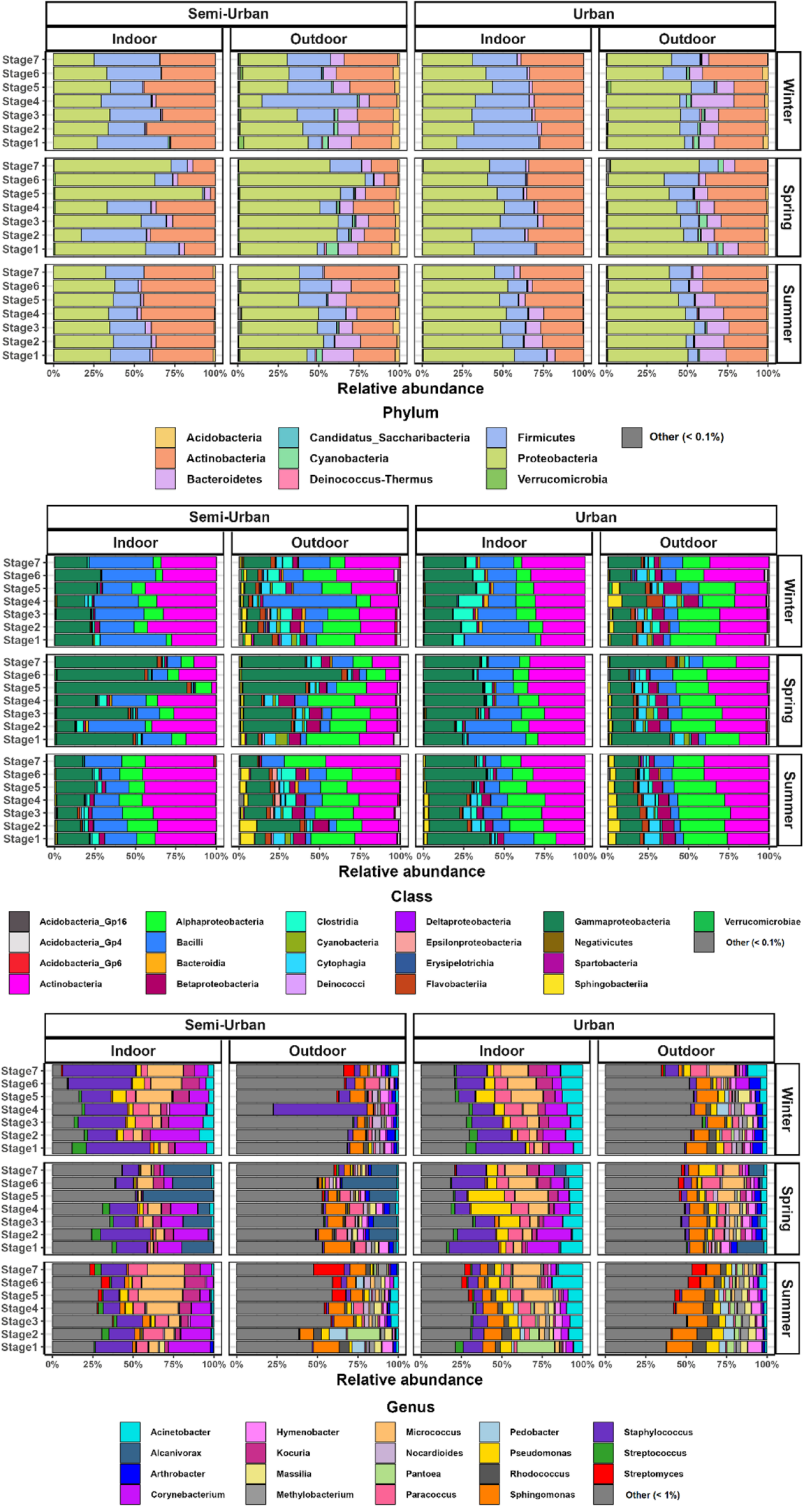
Relative abundance of indoor and outdoor air bacterial OTUs at the phylum, class and genus level per each site (semi-urban & urban) and sampling stage, indoors and outdoors, and across seasons (winter, spring, summer). For phyla/classes “Other” denotes the taxa observed at < 0.1% mean relative abundance, whereas for genera “Other” denotes the taxa detected at < 1% mean relative abundance, across samples. Size-resolved samples were obtained with the May impactor. Stages 1, 2, 3, 4, 5, 6 & 7 correspond to aerodynamic cut-offs of 16, 8, 4, 2, 1, 0.5 and 0.25 μm, respectively.

Overall, Proteobacteria (43%), Actinobacteria (29%) and Firmicutes (18%) were the most abundant bacterial phyla in indoor and outdoor air (across all size fractions) for both residences across the year. (Fig. 2). During winter, similar levels of mean relative proportions (average across all size fractions) were found for the dominant phyla for both sites indoors, despite the substantial differences observed outdoors for Proteobacteria (30.8% for the semi-urban and 43.9% for the urban) and Firmicutes (26.1% for the semi-urban and 11.1% for the urban). It’s worth noting that within members of Firmicutes, although the relative abundance of *Bacilli* was higher (19.8%) in the outdoor air of the semi-urban residence, compared to the urban (7.7%), *Clostridia* were more abundant in indoor air at the urban site (9.2%), compared to the semi-urban house (1.4%).

During spring, Proteobacteria levels were generally higher for both sites, with the most pronounced increase observed for the semi-urban site (51.9% indoors and 59.4% outdoors, compared to 31.1 and 30.8%, respectively, during winter), mostly driven by *Gammaproteobacteria* (46.3% indoors and 31.3% outdoors compared to 22.4 and 8.5%, respectively, during winter). Actinobacteria (18%) and Firmicutes (9%) were noticeably decreased in the outdoor air of the semi-urban residence compared to the winter levels (26 and 26.1%, respectively) and were lower than the levels in the urban outdoor environment (28.9 and 12.1%, respectively). Within Firmicutes in indoor air, the proportion of *Bacilli* appeared decreased (15.3%) in the semi-urban flat and *Clostridia* were substantially reduced (3%) in the urban residence compared to winter levels.

During summer, Proteobacteria were, on average, higher in the urban site (50.1% indoors and 46% outdoors), compared to the semi-urban (35.2% indoors and 42.8% outdoors). Within the class level, *Gammaproteobacteria* levels appeared lower in the indoor and outdoor air of both residences, compared to spring, with the biggest decrease observed for the semi-urban site. However, *Alphaproteobacteria* and *Betaproteobacteria* were both substantially increased indoors, with outdoor levels remaining relatively similar to the ones in spring. It’s worth noting that *Betaproteobacteria* were, in general, more abundant in coarse particles (stages 1–4). The relative proportions of *Epsilonproteobacteria* were noticeably greater outdoors, for the semi-urban site only (1.6% compared to 0.03–0.47% for winter-spring), especially for stages 3–6. Actinobacteria, mainly represented by members of *Actinobacteria* class, were significantly higher in the semi-urban site only indoors (41.5% compared to 28.7% for the urban). Firmicutes (mainly *Bacilli*) appeared decreased only for the urban site (13.8% indoors and 8.4% outdoors, compared to 24.8 and 12.1%, respectively, during spring).

In terms of differences between indoor and outdoor air, Bacteroidetes were consistently found more enriched outdoors compared to indoors, for both residences, with the highest mean relative abundance observed during summer at the urban site. *Gammaproteobacteria* were consistently higher indoors, compared to outdoors, whereas *Alphaproteobacteria* and *Betaproteobacteria* were more enriched outdoors, in both sites. It's also worth noting that several taxonomic groups encountered at lower proportions, including Acidobacteria, Cyanobacteria, Verrucomicrobia and Deinococcus-Thermus, remained consistently more abundant outdoors, compared to indoors, throughout the seasons, indicating an outdoor origin.

At the genus level, overall, *Staphylococcus* (*Bacilli*) was the most abundant taxon found indoors, on average, in both sites, with the highest levels observed in the semi-urban flat during winter. Other dominant genera observed in higher relatively abundance indoors across all seasons included other common human skin commensals such as *Micrococcus* (*Actinobacteria*), *Corynebacterium* (*Actinobacteria*), *Acinetobacter* (*Gammaproteobacteria*) and *Kocuria* (*Actinobacteria*). Among those, *Acinetobacter* was noticeably higher in the urban residence, throughout the year, whereas *Corynebacterium* was higher in the semi-urban residence. In addition, *Micrococcus* and *Kocuria* were more abundant in the fine fraction (stages 5–7), whereas *Corynebacterium* demonstrated increased proportions for the larger particles (stages 1–4). In terms of taxa associated with outdoor environments, of particular interest was the predominance of *Alcanivorax* (*Gammaproteobacteria*) during spring at the semi-urban site, both indoors and outdoors. Size-resolved Indoor-to-Outdoor (I/O) genus relative abundance ratios of the top 10 taxa for each season (Fig.S4) confirmed that taxa encompassing species commonly associated with humans exhibited ratios greater than unity, in most cases exceeding 10, whereas genera with presumptive environmental origins, such as *Sphingomonas* (*Alphaproteobacteria*), *Arthrobacter* (*Actinobacteria*), *Hymenobacter* (*Cytophagia*), showed mostly ratios lower than unity. Interestingly, *Paracoccus* (*Alphaproteobacteria*) and *Pseudomonas* (*Gammaproteobacteria*) were the only environmental taxa that demonstrated I/O ratios > 1 throughout the seasonal cycle (with a few exceptions).

Indicator species analysis (Table S5**–**Table S9) showed that, among airborne genera found at > 1% mean relative abundance across all samples, *Nocardioides* and *Arthrobacter* (winter and spring), *Hymenobacter*, *Rhodococcus* and *Streptomyces* (spring) and *Pedobacter* (all seasons) were strongly associated with the outdoor environment for the semi-urban site. For the urban site, highly abundant taxa indicative of the outdoor environment included *Hymenobacter*, *Rhodococcus*, *Nocardioides* and *Massilia* (winter), as well as *Pedobacter* (spring). In terms of taxa characteristic for the indoor environment, among the genera identified at > 1% mean relative abundance, only *Streptococcus* was found to be a strong indicator of the semi-urban flat interior for summer. Other taxa < 1% which were found strongly associated with the indoor environment in both sites included *Propionibacterium*, *Peptoniphilus*, *Haematobacter* and members of *Clostridia*, *Anaerococcus* and *Finegoldia*, which comprise several human-associated species. In terms of particle size, unique genera associated with fractions < 2 μm or > 2 μm were also investigated. The only indicator taxon > 1% found was *Streptomyces*, which was strongly associated with particles < 2 μm (i.e. stages 5, 6 and 7).

### Size-resolved diversity of bacterial aerosols

**Figure 3:**
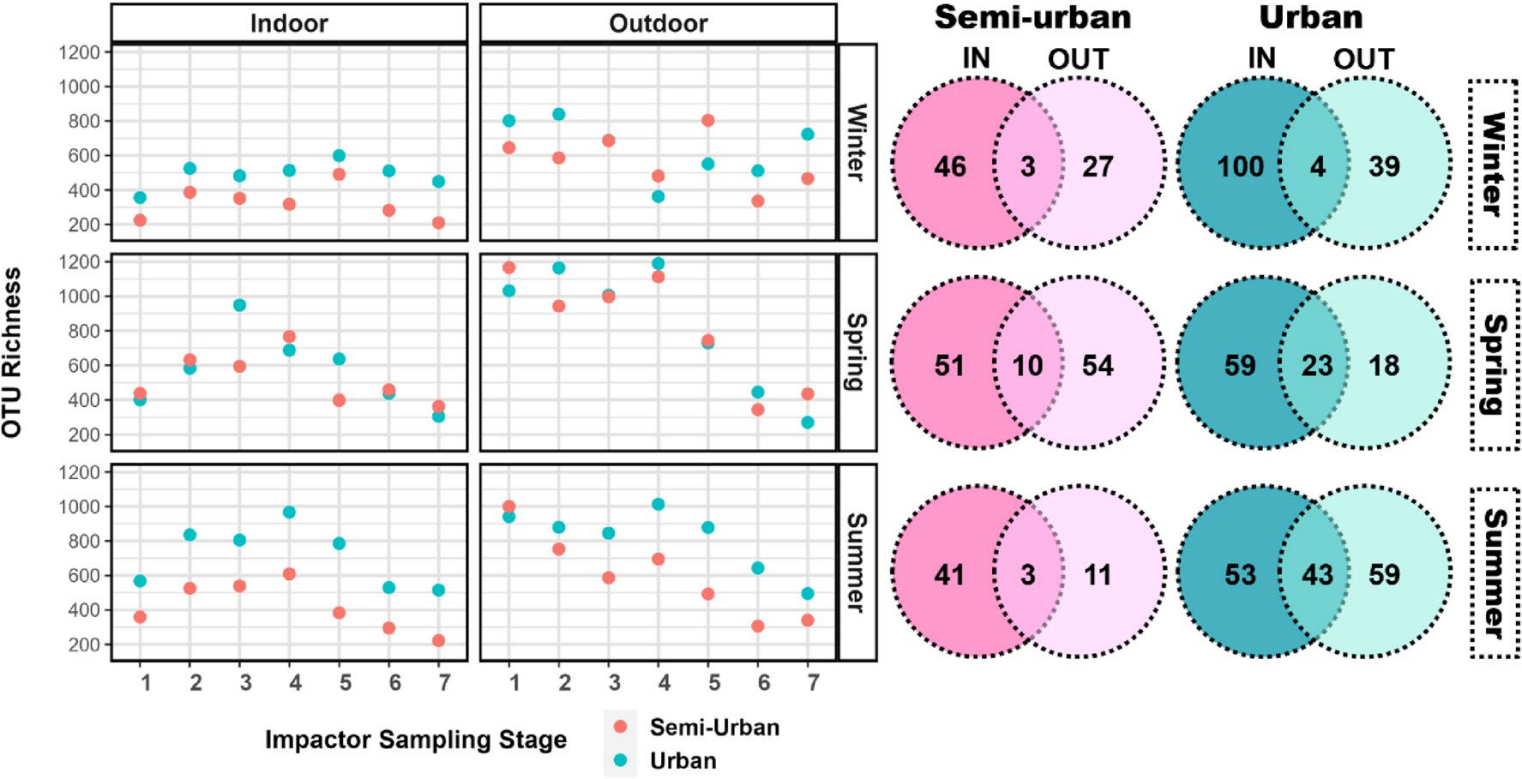
On the left panel: numbers of observed bacterial OTUs, per each sampling site and season, recovered from size-resolved aerosol samples collected indoors and outdoors with the seven-stage May impactor. On the right panel: Venn diagram displaying the number of shared (overlapping regions) and unique bacterial core OTUs (i.e. common OTUs detected across all sampling stages) between indoor and outdoor air samples, per each sampling site and season.

The numbers of bacterial OTUs recovered per each size fraction, as well as core OTUs detected across all size fractions, for both sites, are summarised in Fig. 3. Alpha diversity estimated using Shannon’s Diversity Index is also presented (Fig.S5). Overall, the urban site demonstrated a higher level of bacterial richness, with the difference between the two sites found to be significant only indoors (*t*-test, *p* = 0.002). However, during spring the numbers of OTUs were greater for several size fractions at the semi-urban site. In addition, levels were, overall, significantly higher outdoors, compared to indoors, for both sites (urban: *t*-test, *p* = 0.018, semi-urban: Welch’s *t*-test, *p* < 0.001). Size-resolved OTU richness was significantly associated with 16S rRNA gene abundance (Spearman’s rank, ρ = 0.67, *p* < 0.01), with particles > 1 μm exhibiting greater OTU levels compared to smaller particles (stages 5 & 6).

**Figure 4:**
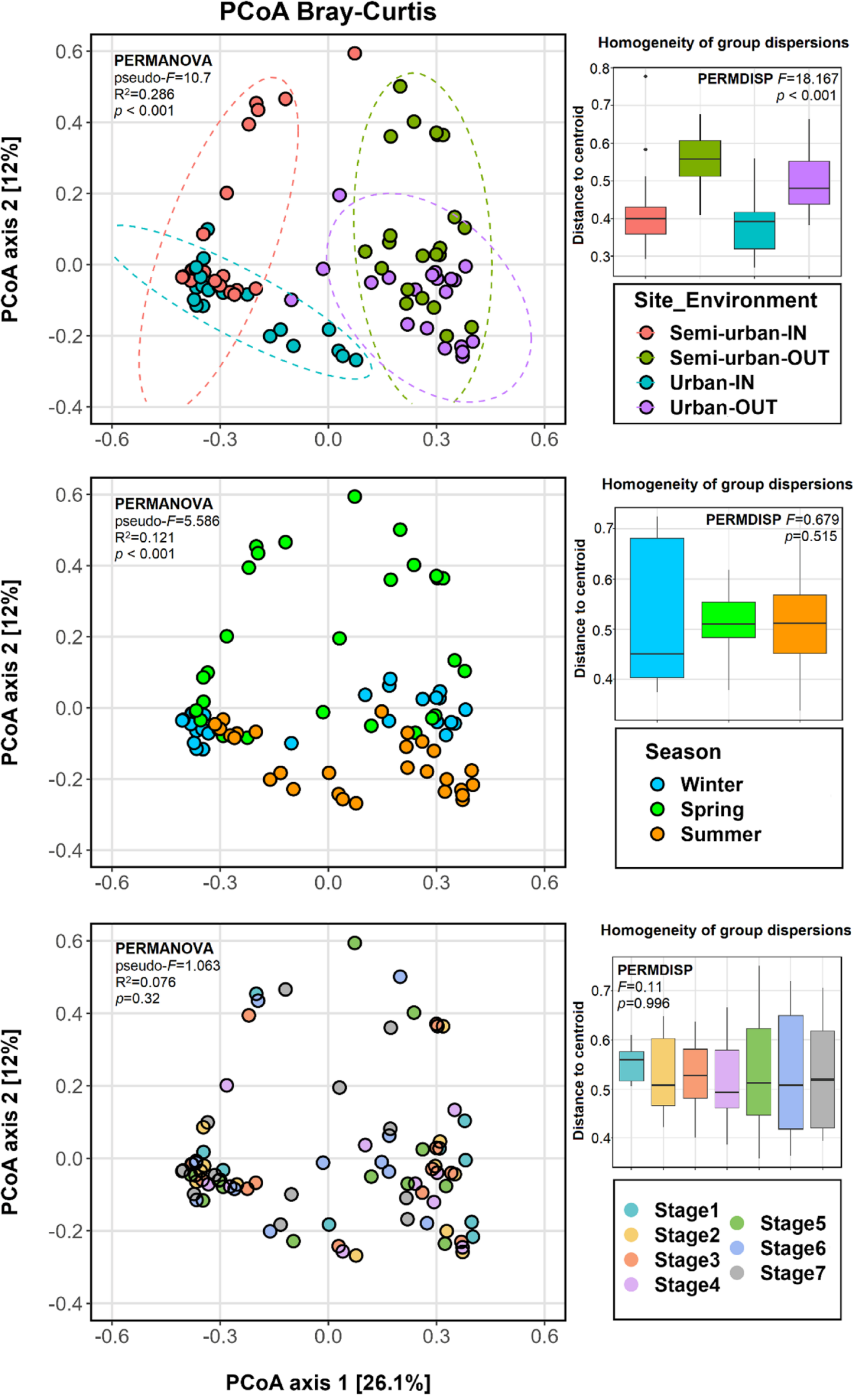
Principal coordinate analysis (PCoA) plots of bacterial beta diversity (left) and boxplots of multivariate homogeneity of group dispersions (right) based on Bray–Curtis dissimilarity with samples grouped (coloured) based on site (urban/semi-urban) and environment (indoor/outdoor), season and sampling stage. Size-resolved aerosol samples were obtained with the seven-stage May impactor. Stages 1, 2, 3, 4, 5, 6 & 7 correspond to aerodynamic cut-offs of 16, 8, 4, 2, 1, 0.5 and 0.25 μm, respectively. Ellipses indicate 95% confidence intervals. Results of PERMANOVA and PERMDISP analysis are also shown.

Beta diversity analysis based on Bray–Curtis was used to investigate the factors driving compositional variation. Ordination results (Fig. 4) showed that samples clustered by both site (urban/semi-urban) and environment (indoor/outdoor). PERMANOVA confirmed that the aerosol bacterial assemblages exhibited significant differences between the two sites (pseudo-*F*_1,82_ = 4.309, R^2^ = 0.04,* p* < 0.001) as well as between indoor and outdoor air (pseudo-*F*_1,82_ = 22.651, R^2^ = 0.216,* p* < 0.001) in both sites (urban: pseudo-*F*_1,40_ = 13.752, R^2^ = 0.256, *p* < 0.001, semi-urban: pseudo-*F*_1,40_ = 12.855, R^2^ = 0.243, *p* < 0.001). Although dispersion values were found to vary significantly between indoor and outdoor samples (PERMDISP, urban: *F* = 25.206, *p* < 0.001, semi-urban: *F* = 21.67,* p* < 0.001), the clustering pattern clearly supported the PERMANOVA results. Seasonality had a significant effect on the overall air microbiome and appeared to be a more important factor (pseudo-*F*_2,81_ = 5.586, R^2^ = 0.121,* p* < 0.001) compared to site, but the effect was smaller compared to environment. The groups of indoor/outdoor samples that were found at closer proximity (i.e., less dissimilar) were the ones obtained during summer for the urban site and the ones obtained during spring for the semi-urban site, whereas the most dissimilar indoor/outdoor samples were obtained during winter for both sites. The particular pattern is also reflected at the Bray–Curtis dissimilarity values (Fig.S6) and PERMANOVA results (Table S10). Samples did not show any clustering according to sampling stage (*p* > 0.05). However, when grouping samples based on coarse (> 2 μm) and fine (< 2 μm) particles, the size fraction was found to have a significant but weak effect on the composition variation (pseudo-*F*_1,82_ = 3.441, R^2^ = 0.04,* p* = 0.001).

**Figure 5:**
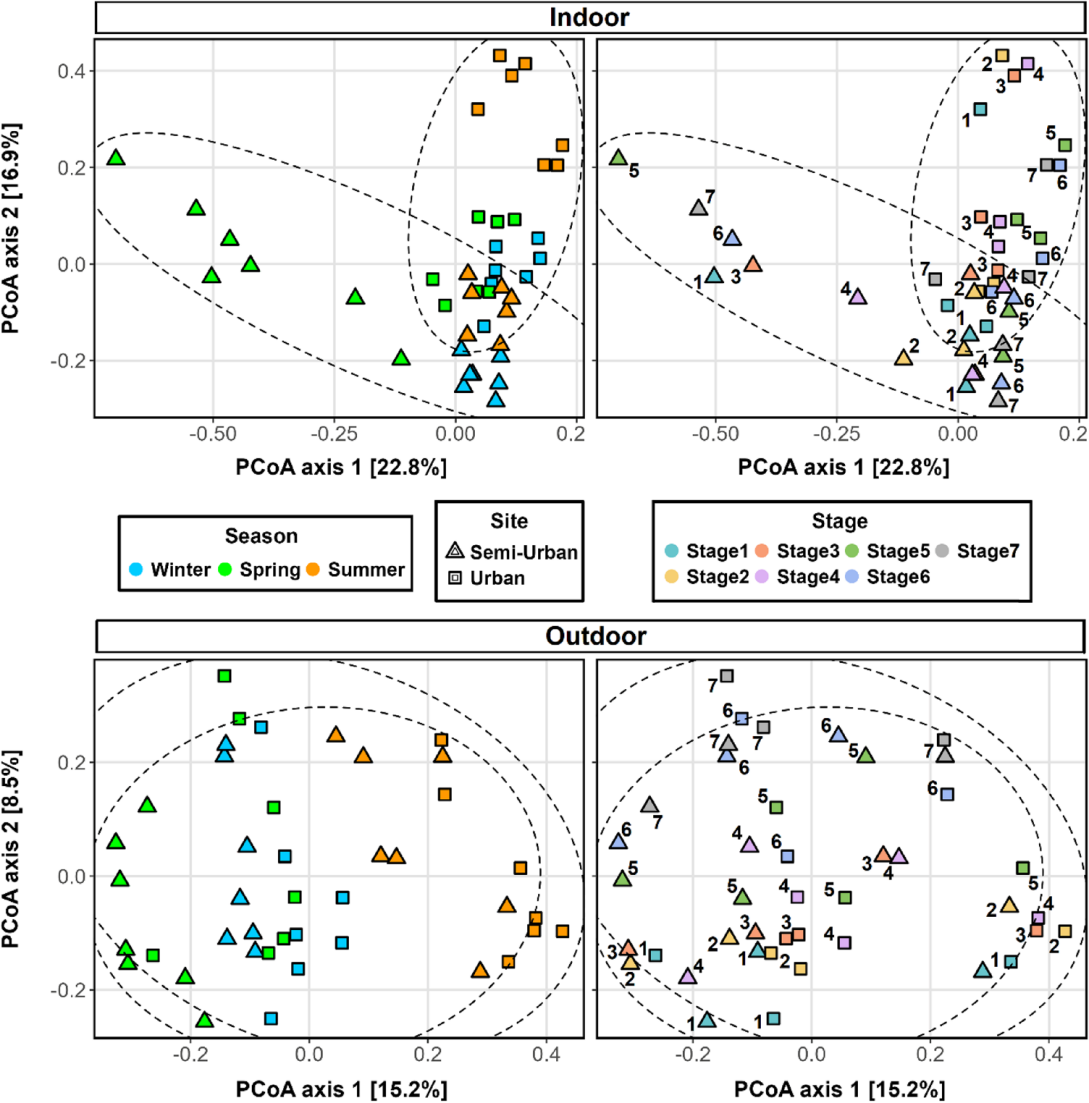
Principal coordinate analysis (PCoA) plots of bacterial beta diversity based on Bray–Curtis dissimilarity for size-resolved aerosol samples obtained indoors (top) and outdoors (bottom). Samples have different shapes based on site (urban/semi-urban). On the left, samples are coloured based on season and on the right, samples are coloured and annotated based on sampling stage. Sets of points corresponding to different sites are also annotated via ellipses.

When examining the indoor and outdoor air bacterial communities separately, PCoA results (Fig. 5) showed distinct groupings based on season rather than site for the outdoor samples, whereas indoor samples clustered according to site. PERMANOVA revealed that seasonality had, in fact, a greater influence on the outdoor aerosol microbiome (pseudo-*F*_2,39_ = 4.581, R^2^ = 0.19, *p* < 0.001), compared to site (pseudo-*F*_1,40_ = 2.335, R^2^ = 0.055, *p* = 0.001). Site appeared to be a more important factor in explaining compositional differences in indoor air (pseudo-*F*_1,40_ = 6.572, R^2^ = 0.141, *p* < 0.001). However, season still explained the most variation indoors both when considering sites together (pseudo-*F*_2,39_ = 5.401, R^2^ = 0.217, *p* < 0.001) and separately (Table S10). The size fraction (> 2 μm/ < 2 μm) was found to have a larger effect in shaping the air microbiome in both sites indoors (urban: pseudo-*F*_1,19_ = 3.78, R^2^ = 0.166, *p* < 0.001, semi-urban: pseudo-*F*_1,19_ = 2.88, R^2^ = 0.132, *p* = 0.02), compared to outdoors (urban: pseudo-*F*_1,19_ = 2.297, R^2^ = 0.108, *p* = 0.006, semi-urban: pseudo-*F*_1,19_ = 1.653, R^2^ = 0.08, *p* = 0.02).

**Figure 6:**
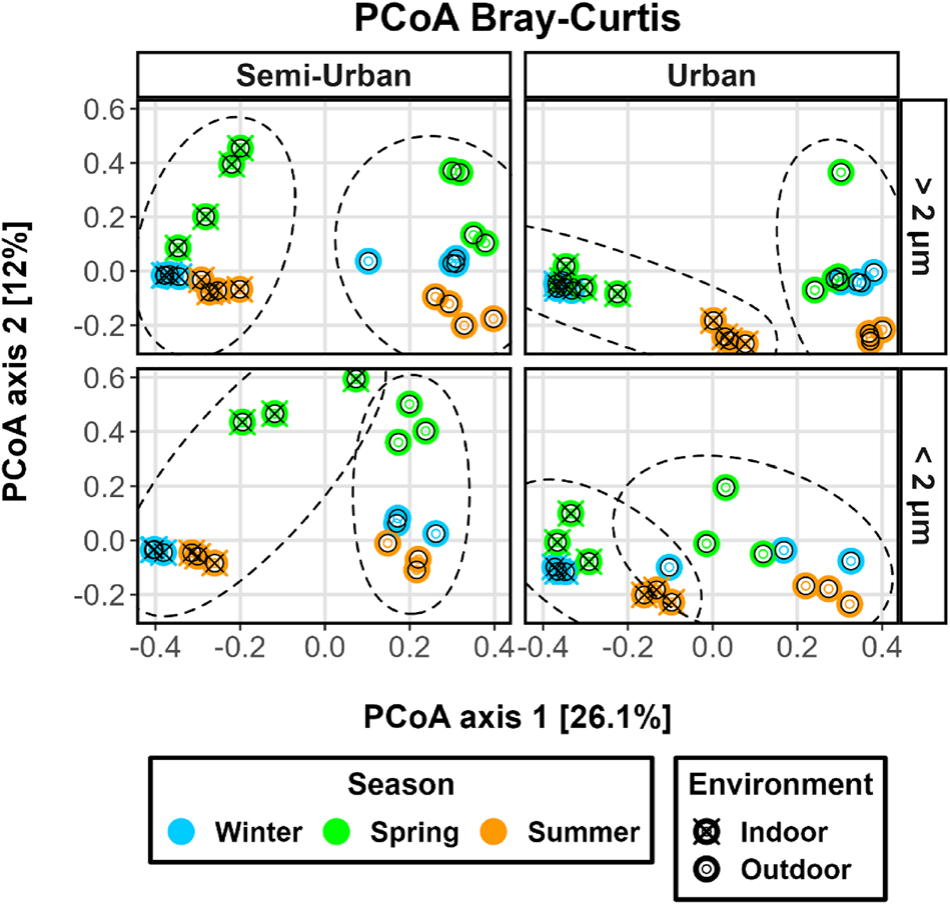
Principal coordinate analysis (PCoA) plots of bacterial beta diversity based on Bray–Curtis dissimilarity for each site (urban/semi-urban). Shape indicates environment (indoor/outdoor). Aerosol samples are coloured based on season and presented based on grouped sampling size bins corresponding to coarse and fine particle size fractions: “ > 2 μm” (i.e. stages 1, 2, 3 & 4) and “ < 2 μm” (i.e. stages 5, 6 & 7), respectively.

The effects of influencing factors were also examined considering the two fractions separately (Fig. 6). For the urban site, ordination plots demonstrated higher similarity between winter and spring samples for both size fractions, indoors and outdoors. The semi-urban site exhibited higher similarity between winter and spring outdoors only for particles > 2 μm, with samples being more similar between winter and summer indoors as well as for both indoor and outdoor particles < 2 μm. In addition, PCoA revealed that the higher indoor-outdoor similarity observed during spring for the semi-urban residence was mostly driven by particles < 2 μm, whereas for the urban site the more similar summer indoor-outdoor samples appeared at relatively similar proximity for both fractions. PERMANOVA indicated that the environment was a greater factor for particles > 2 μm (pseudo-*F*_1,46_ = 19.077, R^2^ = 0.293, *p* < 0.001) compared to particles < 2 μm (pseudo-*F*_1,34_ = 8.167, R^2^ = 0.194, *p* < 0.001). For indoor air, seasonality played a greater role in structuring the microbiome of particles < 2 μm (pseudo-*F*_2,15_ = 3.85, R^2^ = 0.339, *p* < 0.001, > 2 μm: pseudo-*F*_2,21_ = 3.435, R^2^ = 0.247, *p* < 0.001), whereas for outdoor air seasonality had a larger effect on the composition variability of particles > 2 μm (pseudo-*F*_2,21_ = 4.103, R^2^ = 0.281, *p* < 0.001, < 2 μm: pseudo-*F*_2,15_ = 2.198, R^2^ = 0.227, *p* < 0.001).

### Size-resolved source tracking of bacterial aerosols

**Figure 7:**
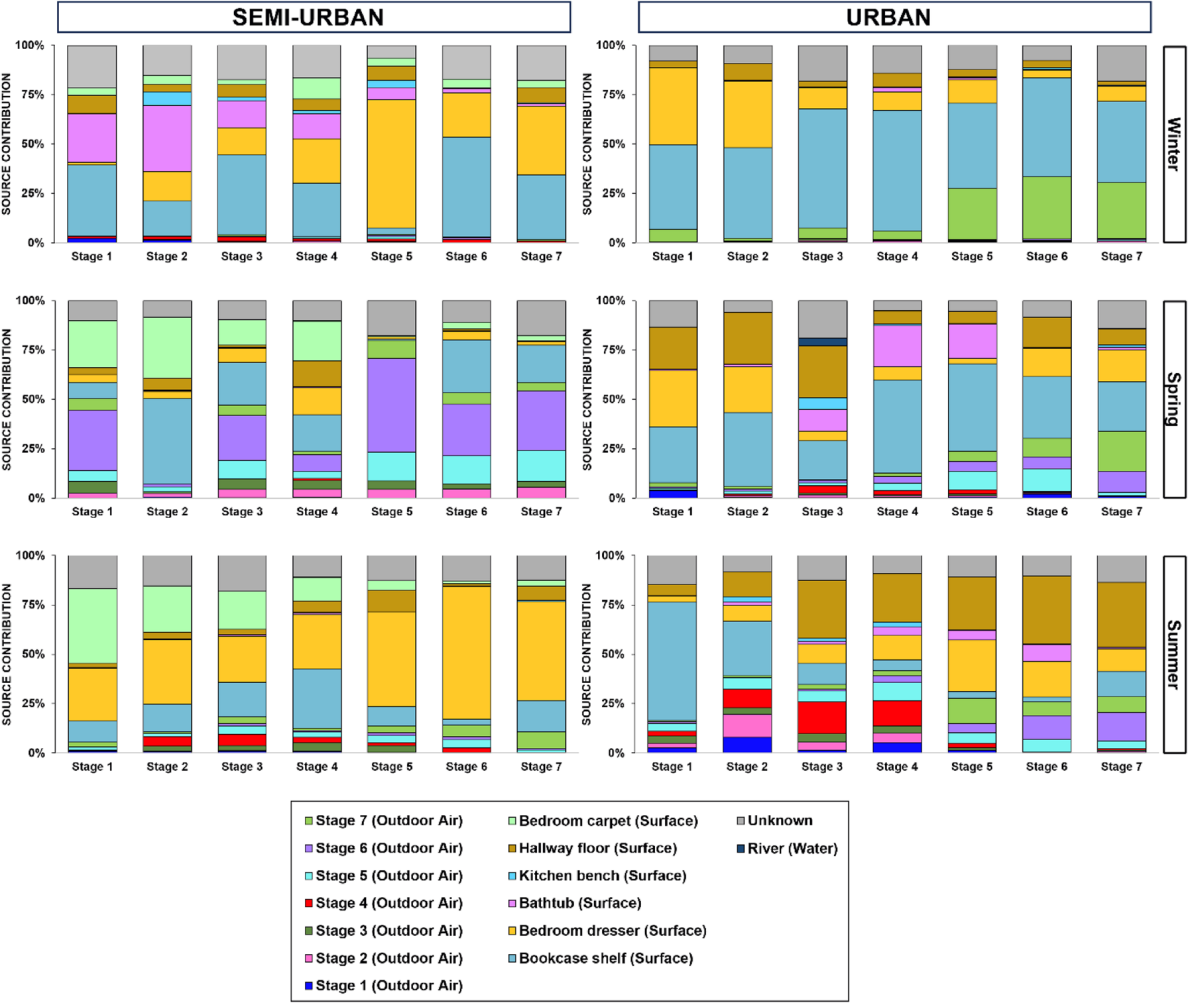
Fast Expectation–mAximization microbial Source Tracking (*FEAST*)-based estimation of the contribution of various sources (size-resolved outdoor aerosols, interior surfaces, river water and unknown sources) to the indoor size-resolved aerosol microbiome for both study sites over seasons.

**Figure 8:**
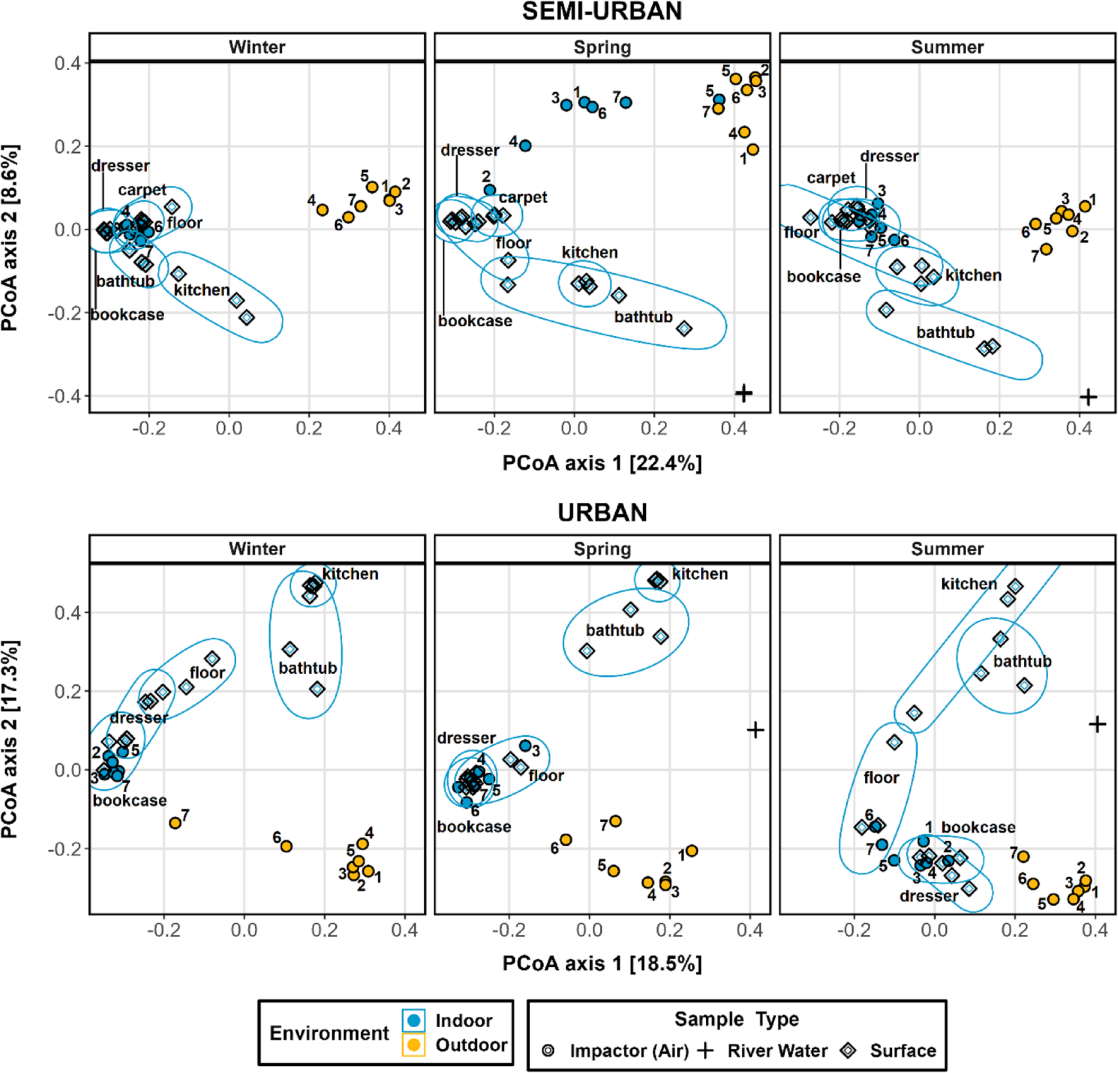
Principal coordinate analysis plots of bacterial beta diversity based on Bray–Curtis dissimilarity for air samples and source-tracking samples, per each site (urban/semi-urban) across seasons. Samples have different colours based on collection environment (indoor/outdoor) and shapes based on sample type: impaction-based (air), surface and water samples collected from the adjacent rivers (river samples were not collected during winter). Impaction-based collected samples are also annotated based on sampling stage. Sets of points corresponding to different types of surface samples are annotated via ellipses.

The relative contribution by potential sources to the size-resolved indoor aerosol microbiome was determined (Fig. 7). Beta diversity ordination results among different types of samples are also presented (Fig. 8) and compositional data for all types of samples are provided (Fig. S7–Fig. S9). Overall, results showed that interior surfaces, which were found to vary significantly in terms of composition between the two sites and across seasons (Fig. S10 and Table S11), presented the greatest source proportion, surpassing that of outdoor air, except for the semi-urban house during spring (average contribution of 45.75% from outdoor air compared to 42.09% from the surfaces) (Fig.S11). It’s worth noting that the high contribution of outdoor air determined for the semi-urban indoor bioaerosols (all size fractions except stage 2) was mostly driven by fine particles. The highest contribution from outdoor air for the urban residence was observed during summer (average contribution of 30.65% from outdoor air compared to 58.04% from the surfaces), with outdoor particles > 1 μm dominating the larger size fractions (stages 1–4) in indoor aerosols and particles < 2 μm being the main outdoor air source for the corresponding smaller size fractions (stages 5–7) indoors. In terms of surfaces, the microbiome of the bookcase shelf (living room) and dresser (bedroom), which were found to be compositionally similar in both sites (Tables S12**, **S13) and dominated by taxa similar to the ones found in indoor air, accounted for the highest contribution, overall, in varying proportions across size fractions and seasons. The contribution from the hallway floor (urban) and bedroom carpet (semi-urban site) microbiota appeared increased during spring and summer. Finally, the water microbiome from the adjacent rivers was found to have a negligible contribution (< 1%) to the indoor air microbiome at both sites.

## Discussion

This study investigated the bacterial abundance and diversity in size-resolved fractions of indoor bioaerosols in urban and semi-urban residences, in parallel with the outdoor bioaerosols, in the UK, throughout the year. Our findings demonstrated that there was a greater abundance of bacteria (based on qPCR analysis) in particles > 1 µm but also dispersed across multiple sizes ranges, with maximum levels for the ranges between 1–2 μm and 2–4 μm indoors and > 8 μm outdoors. Our results are in agreement with previous findings showing that for bacteria-laden particles the size distribution in the air is mostly not represented by the size ranges of their cells/spores and bacteria tend to attach on big particles (Clauβ, 2015). Previous qPCR-based investigations have found that the highest concentrations occurred in the coarse fraction, based on sampling using a Harvard high-volume cascade impactor in a suburban house, indoors and outdoors^39^, in the 3.3–4.7 μm and 4.7–7 μm ranges, based on samples collected with a six-stage non-viable Andersen in classrooms^40^, and in the 3.3–4.7 μm range, based on collection with an eight-stage non-viable impactor in a university classroom^20^. The occurrence of bacteria aggregated or adhered to large-sized particles in outdoor air has been reported to provide a protection to the micro-organisms from harsh environmental conditions^41^. Determination of large bioaerosols is important as particles may be fragmented upon deposition and get re-aerosolised as smaller aerosols, capable of entering deeper into the human respiratory system. It must be noted, however, that particle bounce in the impactor may potentially bias the reported size distributions recovered.

The higher indoor air bacterial concentrations compared with outdoors (i.e. I/O ratio greater than 1) for most size ranges during winter and spring suggests that the origin of those particles was most likely associated with in-house sources, whereas the shift of the I/O ratio towards values closer or lower to unity observed in summer indicates that the impact of indoor sources was less pronounced. Owing to the higher air exchange rates typically occurring during summer in naturally ventilated spaces, the indoor concentrations tend to track the outdoor levels more closely. Overall, I/O ratios < 1, denoting a potential outdoor origin, were observed for the largest particles, whilst bacterial aerosols that exhibited the highest ratios were within the fine fraction for both residences. In addition, the shift of the indoor concentration peak from 1–2 μm in winter to 2–4 μm in summer, observed for both houses, suggests a potential mixing with the outdoor air large-sized particles.

In terms of bacterial composition, the particle size fraction played a weak but significant role in structuring the microbiome of fine (< 2 μm) and larger (> 2 μm) aerosols, with bacterial taxa dispersed across all examined sizes. The environment and site (location) combined appeared to be the major drivers of community structure, with the variation occurring between indoors and outdoors more pronounced than the variation between the two residential settings, indicating that the key determinants shaping the indoor and outdoor air microbiomes are different. In addition, the compositional differences between indoor and outdoor aerosols were mostly influenced by the larger particles. Despite the significant differences in bacterial diversity (how many types of bacteria are present) found in indoor air between the two residences, outdoor aerosols exhibited similar levels of bacterial richness. Although the two study sites were characterised by different levels of urbanisation, the aerosol bacterial communities outdoors were found to vary less compared to indoors, most likely related to the fact that the spatial scale was not large enough to provide a location-specific pattern. In addition, the examination of more residential flats in each location (urban/semi-urban) would be required to allow for the evaluation of the spatial dynamics.

The two flats chosen to be sampled in the current study were as similar as possible in terms of various parameters known to structure the indoor microbiome including features such as ventilation type, occupancy patterns, as well as the external environment in the immediate vicinity of the housing buildings. This suggests that the factors contributing to the variation in aerosol microbiomes between the two residences, which was more pronounced for the fine particles, were driven by in-house sources. Indeed, airborne bacteria were found to be predominantly sourced from residential surfaces in both flats. Household surfaces harbour diverse microbial communities which could be a result of either transfer through contact or deposition of microorganisms that were previously airborne^42,43^. Settled microbes can re-enter the air due to resuspension of dust particles induced by indoor activities and thus surfaces (e.g., furnishing and flooring) can act as secondary sources^44,45^. Previous studies have demonstrated that humans leave a distinct microbial signal in the environments they occupy on both surfaces and in the surrounding indoor air^46,47^. Although various common human-associated taxa (e.g., *Staphylococcus*, *Micrococcus*, *Clostridia*) were found to dominate aerosols in both houses, several of those exhibited substantial different relative proportions, which could be linked to the occupants’ personalised microbiome. For instance, *Acinetobacter* was more abundant in the air of the urban flat and was also found to be a lot more enriched on the urban residence surfaces, whereas *Corynebacterium* and *Streptococcus* were detected in noticeably higher abundance in both the air and the surfaces of the semi-urban flat.

*Paracoccus*, known to typically inhabit a wide variety of outdoor environments, was also among the predominant airborne bacteria found to be more enriched indoors for both sites. The particular taxon was detected on furniture surfaces (bookcase, dresser) and the floor in both houses, too, in agreement with previous dust-associated microbiome investigations^48,49^. Moreover, *Pseudomonas*, which is ubiquitous in natural settings, exhibited mostly larger relative proportions in indoor air. Interestingly, *Pseudomonas* was also found on the bathtub surfaces sampled, in line with studies showing that *Pseudomonas* spp*.* colonise plumbing/water distribution systems and toilet bowls, and can be, therefore, aerosolised and settle on surrounding surfaces^50,51^. Both *Paracoccus* and *Pseudomonas* comprise skin-associated species^52^, too, and their presence in the residential air and surfaces could be also the result of skin shedding. It has to be noted, though, that the microbiome in indoor environments is also influenced by the occupants’ behaviour, such as cleaning frequency. In addition, micro-organisms can be carried inside by the occupants via footwear or clothing, followed by subsequent resuspension. For example, outdoor environment-associated taxa such as *Alcanivorax*, *Microbulbifer*, *Colwellia* and *Pseudophaeobacter*, which were highly abundant during spring in the semi-urban residence, exhibited I/O ratios > 1 indicating an indoor origin, which could not be specified. It is also possible that observed differences between the two residences could be related to the presence/absence of indoor plants (e.g. the urban flat had two houseplants). Nevertheless, this could not be determined since plant-related samples were not included in our source-tracking analysis.

The aerosol bacterial communities exhibited significant compositional variation across seasons, both indoors and outdoors, with seasonality being a stronger predictor of community structure compared to site. The seasonal variation observed outdoors is in line with previous HTS-based long-term monitoring surveys of outdoor bacterial aerosols (e.g.,^12,53^). In our study, the seasonal factor for the outdoor environment had a larger effect on particles > 2 μm. It has been previously reported that large bioparticles in the atmosphere show strong seasonal variations due to their dependence on the plant phenology, whereas the abundance of smaller bioaerosol particles (< 2 μm) exhibit minimal variation^54,55^.

Results to date have pointed out that the seasonal dynamics of the outdoor airborne bacteria might not necessarily be reflected indoors, mainly attributed to the dominance of human occupancy-associated bacteria, which have a homogenising effect on bacterial aerosols typically observed over time in enclosed spaces^8,56, 57^. Despite the strong contribution of the residents as a source for bacteria in the current study, one major parameter that contributed to the distinct seasonal differences, indoors, was the natural ventilation occurring throughout the year in both houses, which has been shown to increase the similarity between indoor and outdoor air community composition^7,9^. Naturally ventilated buildings cannot limit the influx of outdoor coarse particles, which carry a more distinct seasonal signal.

The overall predominance of Proteobacteria, Actinobacteria and Firmicutes identified in particles in both sites is in line with previous residential studies^8,58, 59^ and has been consistently observed in the air of both indoor (e.g.,^5,57^) and outdoor environments (e.g.,^11,25^). Similar to our investigation, previous studies, that examined both indoor and outdoor bioaerosols in dwellings^8^ and childcare facilities^10^, demonstrated that the bacterial composition indoors was distinct from the one in outdoor air due to the larger input of human-associated bacteria. In our study, the compositional dissimilarity between indoor and outdoor bacterial aerosols was higher during winter that windows mostly remain shut. In contrast, a pronounced increase of bacteria indicative of the outdoor environment was observed in indoor air during the warmer periods, when natural ventilation rates are highest, including taxa such as *Rhodococcus* and *Hymenobacter*, typically recovered from soil and aqueous environments, *Streptomyces*, which is abundant in soil, and *Pedobacter*, also related with soil and sediments, reflecting the stronger influence of outdoor sources. Several environmental taxa found increased in both sites during summer, such as *Alphaproteobacteria*, *Betaproteobacteria, Cytophagia,* have been previously detected in river water and freshwater systems (e.g.,^60^). However, despite the fact that both study flats were situated in riverside blocks, source tracking analysis estimated that the river water accounted for a very low contribution. Bacteroidetes, which were found outdoors throughout the year, also became distinctly increased in the air of the urban house in summer. The occurrence of Bacteroidetes, which generally comprise gut mammalian microbiota, in outdoor air in cities has been previously linked to dog faecal material^61^.

It should be noted that the two sites exhibited different seasonal patterns, with the highest degree of similarity between indoor and outdoor aerosol microbiomes observed in summer for the urban site and in spring for the semi-urban site. The particular patterns were also reflected in the increase of the contribution of outdoor air as a source during summer and spring for the urban and semi-urban houses, respectively, compared to winter. Although establishing a clear temporal pattern is residence-dependent, due to the impact of house-specific sources and ventilation patterns, this difference might be the result of intra-season variability as sampling for each residence took place on different dates per each season. Within-season community variability could be also related to the local plant phenological periods, as noticed by Weikl et al.^62^, which could be easily overlooked due to limited time-point samples per season. In addition, seasonal variation associated with large-sized particles, which, in our study, exhibited higher bacterial richness, might be also related to the presence of pollen grains in the air^55^, which may act as carriers of highly diverse bacterial communities^63,64^.

## Conclusions

This study aimed to investigate the size-resolved fractions of airborne bacterial communities in two naturally ventilated residential settings, characterised by different levels of urbanisation, by monitoring both indoor and outdoor bioaerosols over a seasonal cycle. Results showed that the indoor and outdoor aerosols harboured distinct bacterial populations in the two residential sites, due to the dominance of human-associated taxa in indoor air, and exhibited significant temporal variation throughout the year. *Actinobacteria*, *Gammaproteobacteria* and *Bacilli* were, on average, the predominant bacterial groups identified indoors, whereas *Actinobacteria*, *Alphaproteobacteria* and *Gammaproteobacteria* were the most abundant taxa outdoors. Our findings demonstrated the importance of both indoor occupancy-associated sources and outdoor air in structuring the bacterial composition of aerosols in residences, with seasonality playing a major role in shaping the relationship between indoor and outdoor aerosol microbiomes.

Overall, bacterial taxa identified were dispersed across particles of various sizes, with the highest concentrations observed for the 1–2 μm and 2–4 μm ranges indoors and > 8 μm outdoors. Although the compositional differences between the two residences were mostly driven by fine particles (< 2 μm), the variation between indoors and outdoors were mostly influenced by larger particles (> 2 μm). In terms of temporal changes, seasonality played a greater role in structuring the microbiome of particles < 2 μm indoors, whereas for outdoor air seasonality had a larger effect on particles > 2 μm. The differences found in the size effect of influencing factors between fine and larger particles highlights the significance of including size distribution data in bioaerosol microbiome investigations. Therefore, further research is required to better understand the role of particle size in shaping the aerosol microbial communities, which is crucial for human exposure assessment.

### Supplementary Information

Supplementary Information.

## Data Availability

All sequence datasets generated and analysed in this study have been deposited into NCBI BioProject database and made available under the accession number PRJNA1078868. All other data generated or analysed during this study are included in this published article (and its Supplementary Information file).
