# Supplementary material for: Temporal changes in the size resolved fractions of bacterial aerosols in urban and semi-urban residences
Source: Sci Rep. 2024 Aug 30;14:20238. doi: 10.1038/s41598-024-70495-3 (PMC11364847; doi:10.1038/s41598-024-70495-3)
Supplement: Supplementary file 1 — Supplementary Information. [file 41598_2024_70495_MOESM1_ESM.docx]

**Supplementary Information**

**Temporal changes in the size-resolved fractions of size-resolved bacterial aerosols in urban and semi-urban residences**

Grydaki N.^1^, Colbeck I.^1^ and Whitby C.^1*^

^1^School of Life Sciences, University of Essex, Colchester CO4 3SQ, Essex, UK

***Corresponding Author:** [cwhitby@essex.ac.uk](mailto:cwhitby@essex.ac.uk)

**Supplementary Figures**


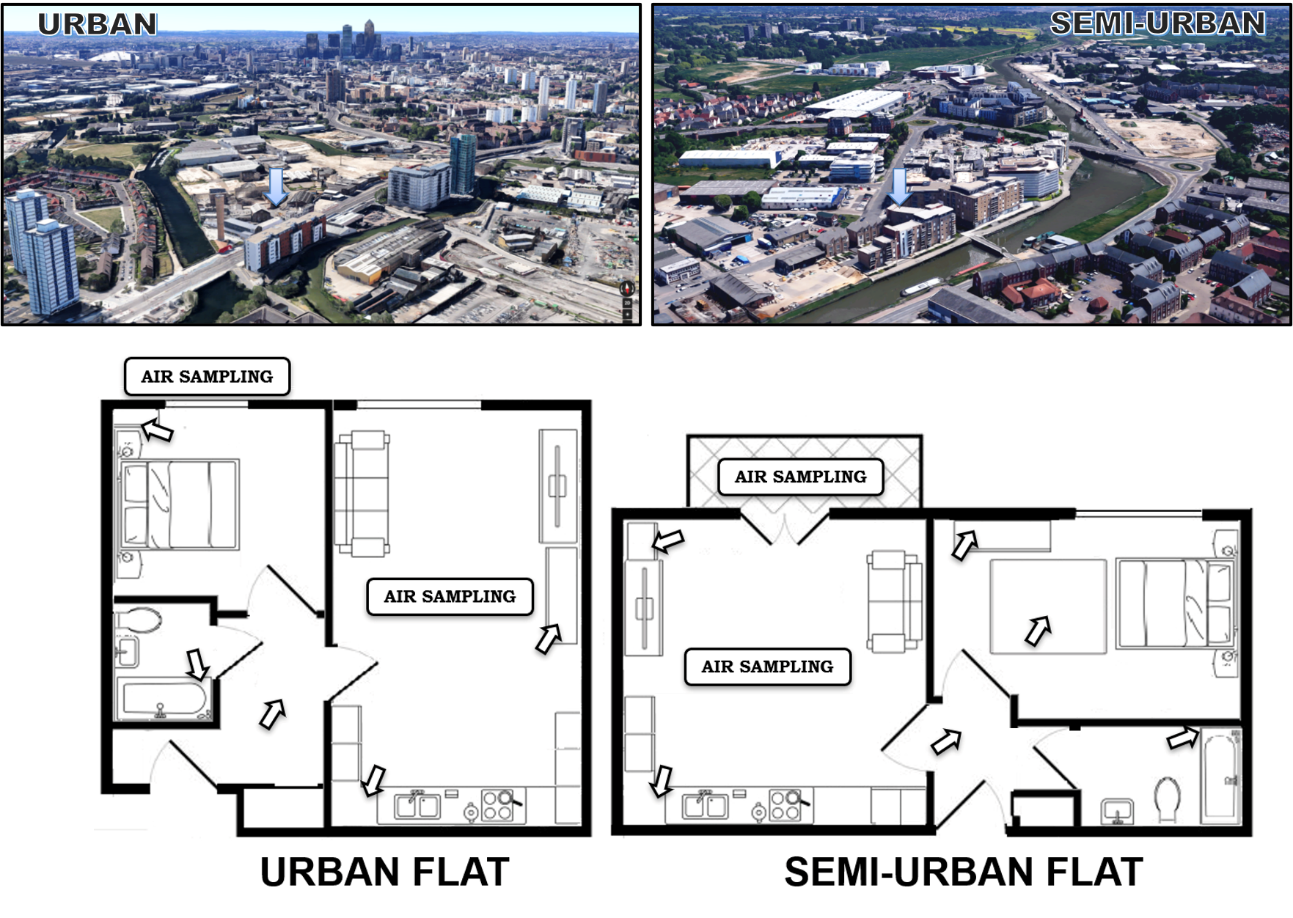


**Figure S1.** Aerial views (3D Google images) (**top**) illustrating the locations of the study houses (residential blocks are indicated by blue arrows) and the surrounding areas in Stratford (London) -urban- and Colchester (Essex) -semi-urban- sites. Floor plans of the study houses (**bottom**) with the indoor and outdoor air sampling locations. The surfaces sampled in each residence: bookcase shelf, kitchen bench, bathtub (tub abutment), bedroom dresser (chest of drawers), hallway (wooden) floor and carpet (only at the semi-urban apartment), are denoted by arrows.


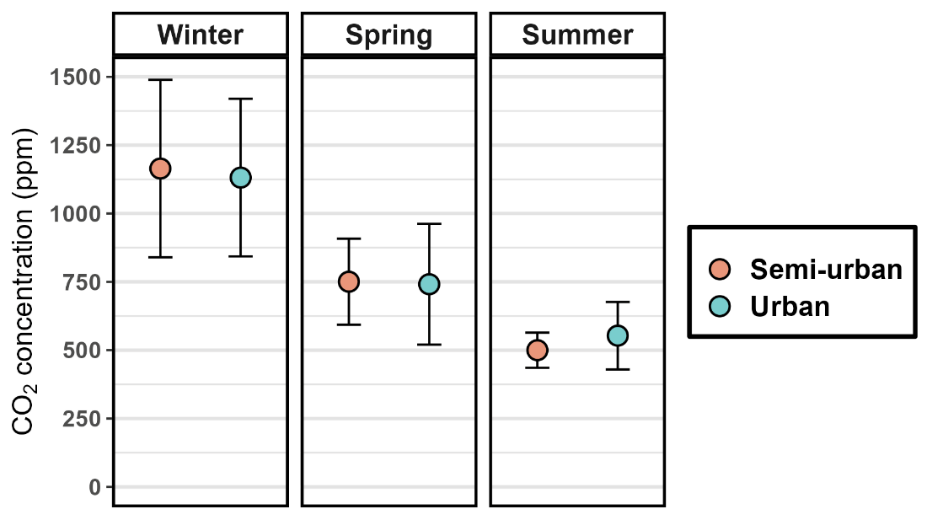


**Figure S2.** Carbon dioxide levels (ppm) measured in the two study residences across seasons. Error bars represent standard deviation of the mean (*n* = 72).


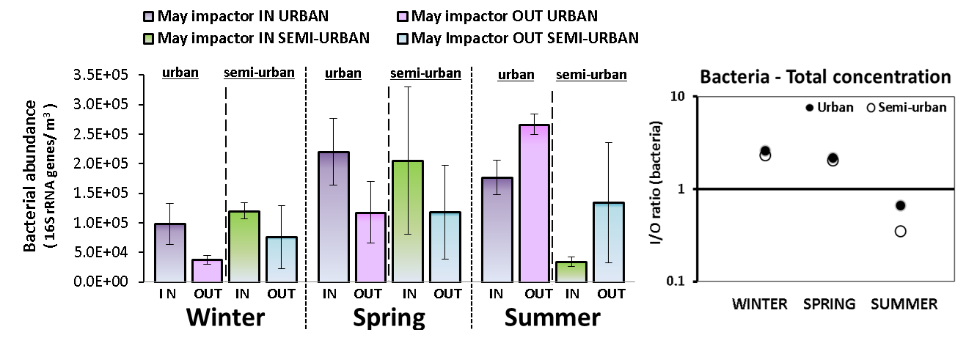


**Figure S3. On the left panel:** Seasonally averaged total concentrations (*n* = 3 days) of aerosol bacterial abundance determined by qPCR (16S rRNA genes per m^3^ of air), indoors and outdoors, for each site. The urban site exhibited significant differences across seasons indoors (ANOVA, *F*_2,6_ = 6.70, *p* = 0.029) and outdoors (ANOVA, *F*_2,6_ = 39.82, *p* < 0.001). However, the season was not found to have any significant effect on the aerosol bacterial abundance indoors (*F*_2,6_ = 4.17, *p* = 0.073) or outdoors (*F*_2,6_ = 0.42, *p* = 0.677) for the semi-urban site. **On the right panel:** Indoor-to-Outdoor concentration ratios (I/O) for airborne bacteria in the urban (closed circles) and the semi-urban (open circles) residences across seasons. Each I/O ratio presented per season is an average over three days.

**
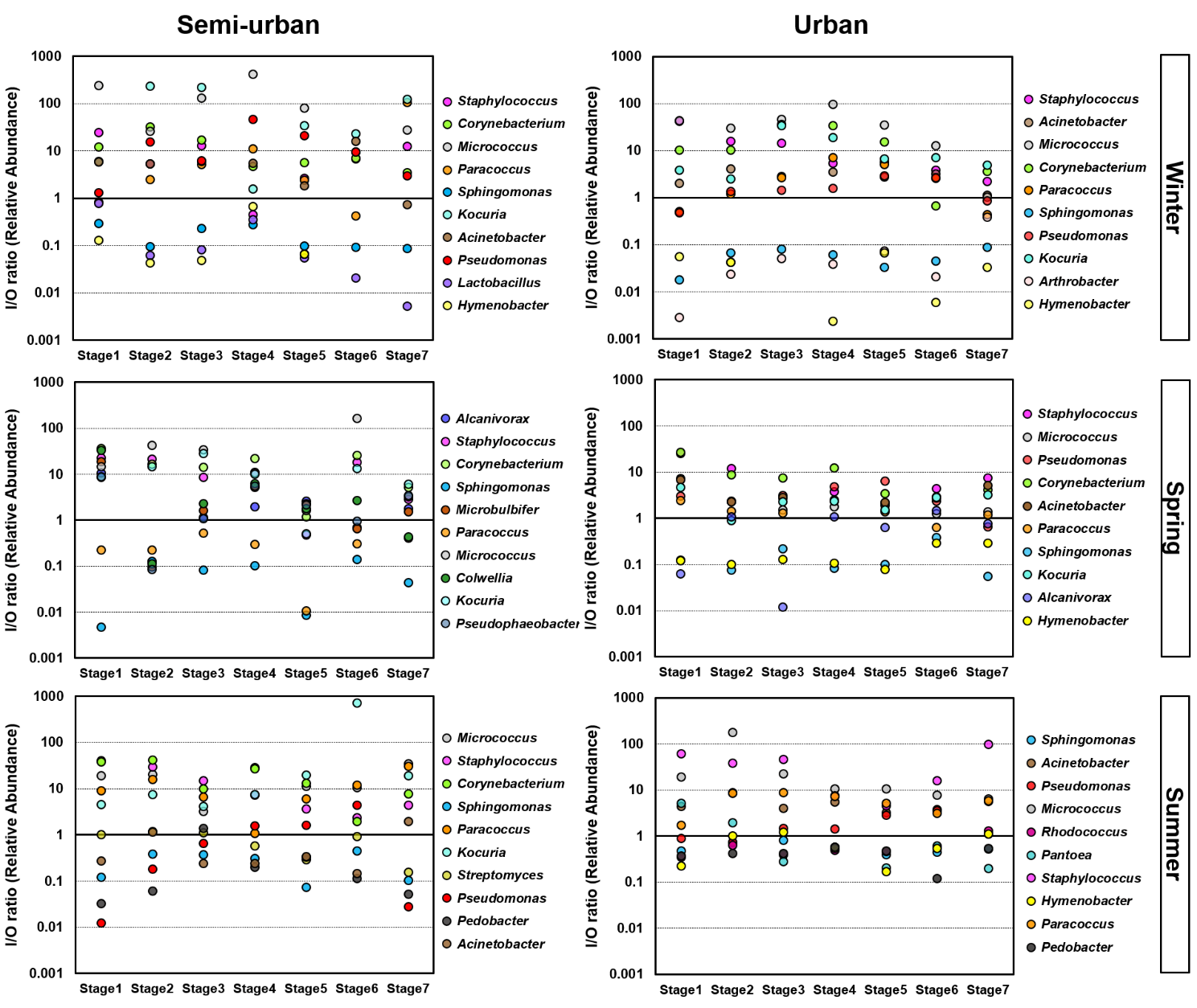
**

**Figure S4.** Size-resolved Indoor-to-Outdoor ratios (I/O) for bacterial genus relative abundance for the semi-urban (**left**) and urban (**right**) sites across seasons. Axis y is on log scale. Axis x represents the May impactor size bins 1, 2, 3, 4, 5, 6 & 7 corresponding to aerodynamic cut-offs of 16, 8, 4, 2, 1, 0.5 and 0.25 μm, respectively. Genera in the legend are ordered according to overall average abundance across indoor and outdoor samples, from most abundant (top) to least abundant (bottom).

**
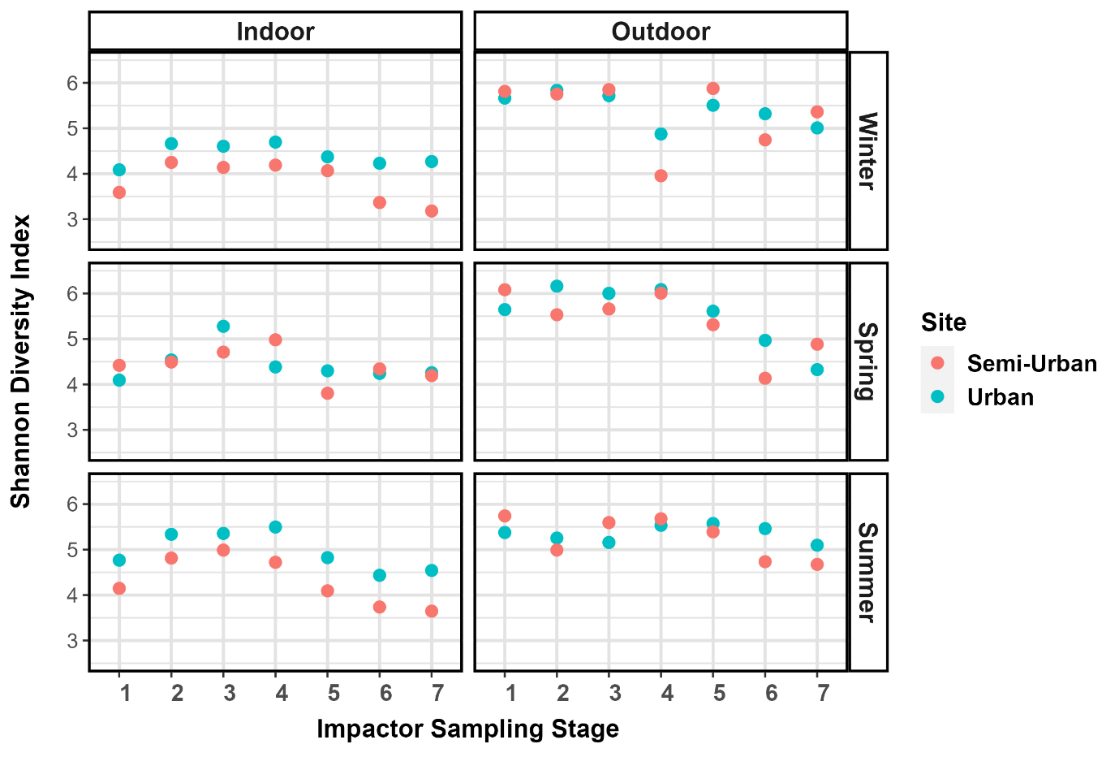
**

**Figure S5.** Shannon’s Diversity Index for size-resolved aerosol samples collected indoors and outdoors with the seven-stage May impactor, per each sampling site and season.

**
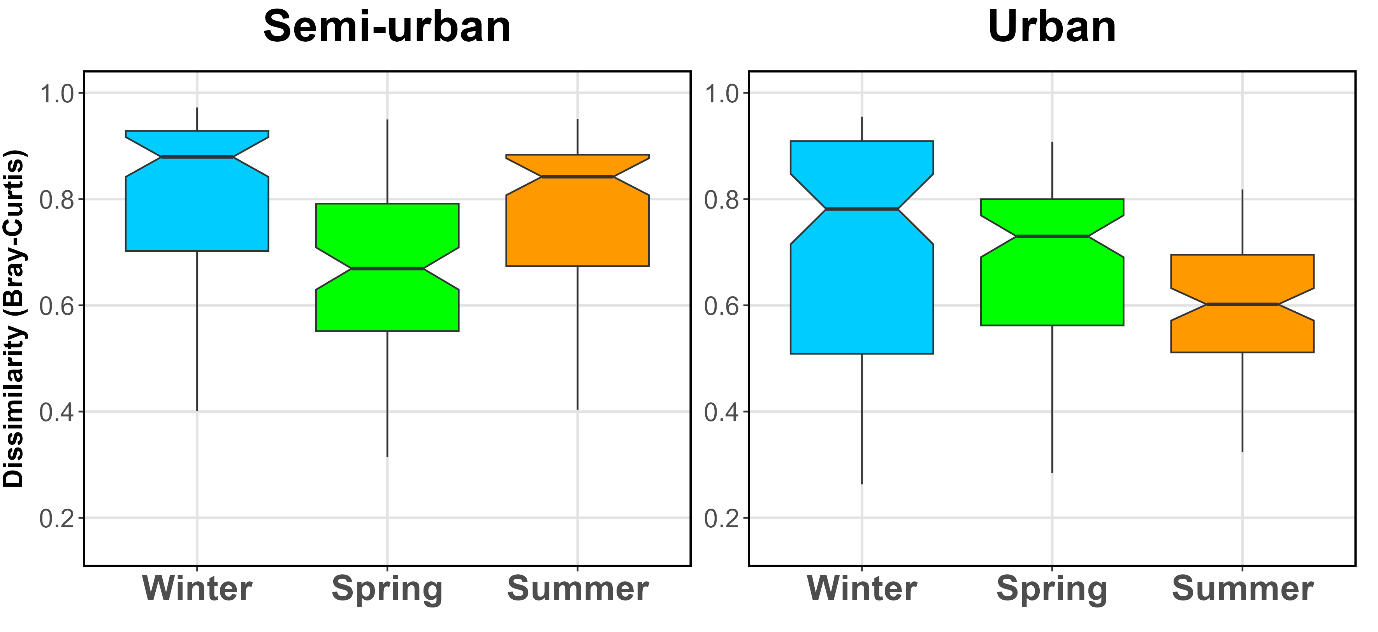
**

**Figure S6.** Boxplots of pairwise Bray-Curtis dissimilarity of aerosol bacterial composition based on both indoor and outdoor air samples within each season and per each site. The Bray-Curtis dissimilarity metric assumes values between 0 and 1, with two samples being identical when the value is 0 and completely dissimilar if the value is 1.


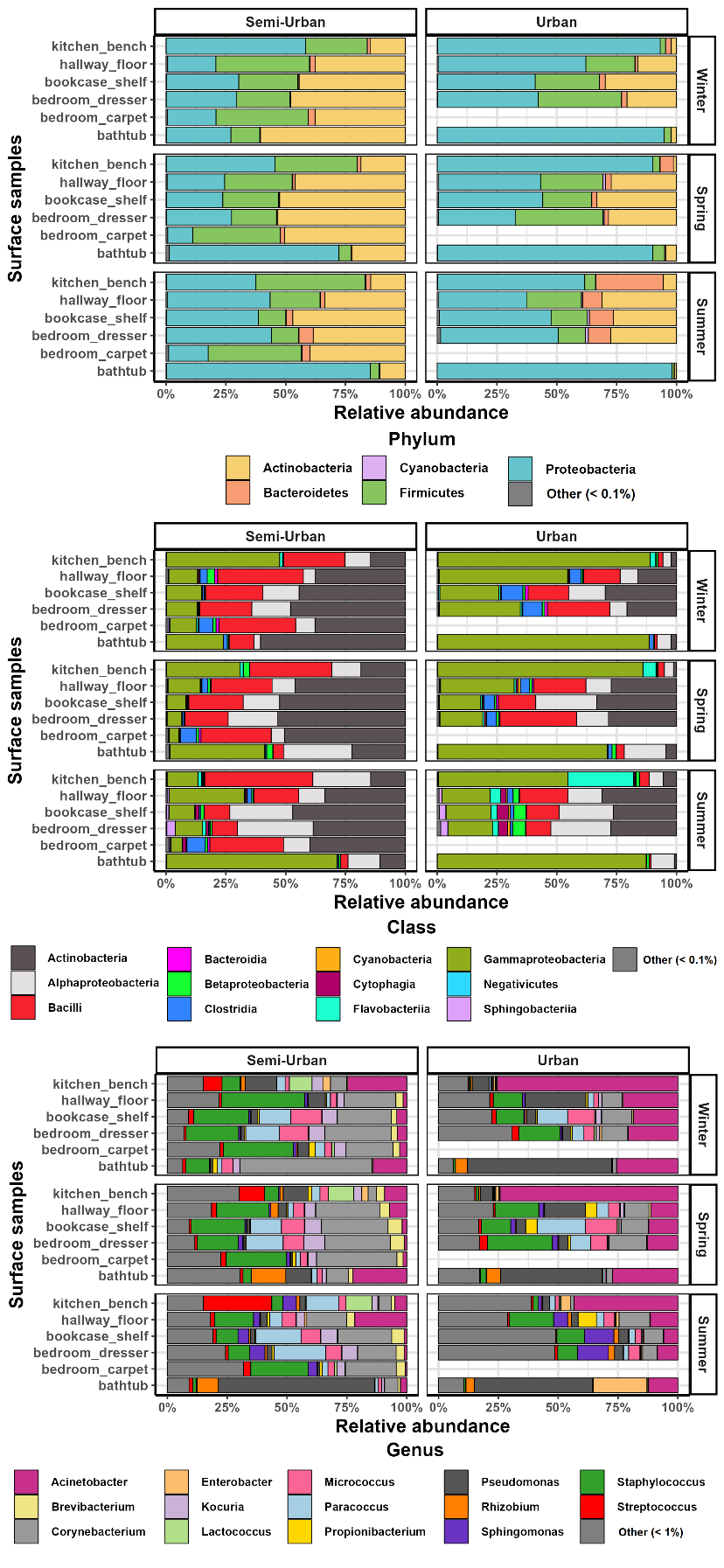


**Figure S7.** Relative abundance of interior surface bacterial OTUs at the phylum, class and genus level per each sampling site (semi-urban & urban), across seasons (winter, spring, summer). For phyla/ classes “Other” denotes the taxa observed at <0.1% mean relative abundance, whereas for genera “Other” denotes the taxa detected at <1% mean relative abundance across samples.


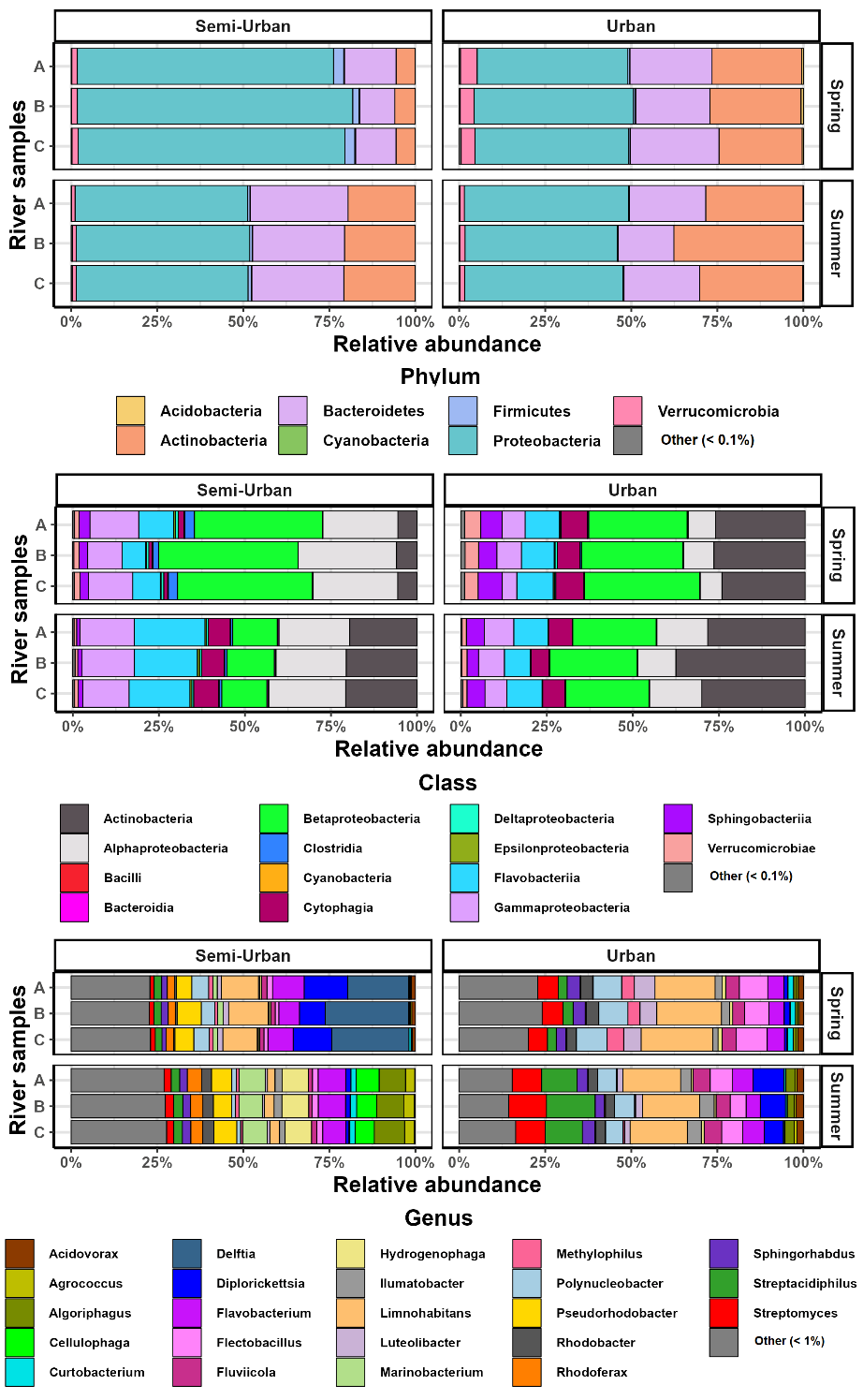


**Figure S8.** Relative abundance of river water bacterial OTUs at the phylum, class and genus level per each sampling site (semi-urban & urban), across seasons (spring & summer). For phyla/ classes “Other” denotes the taxa observed at <0.1% mean relative abundance, whereas for genera “Other” denotes the taxa detected at <1% mean relative abundance across samples.


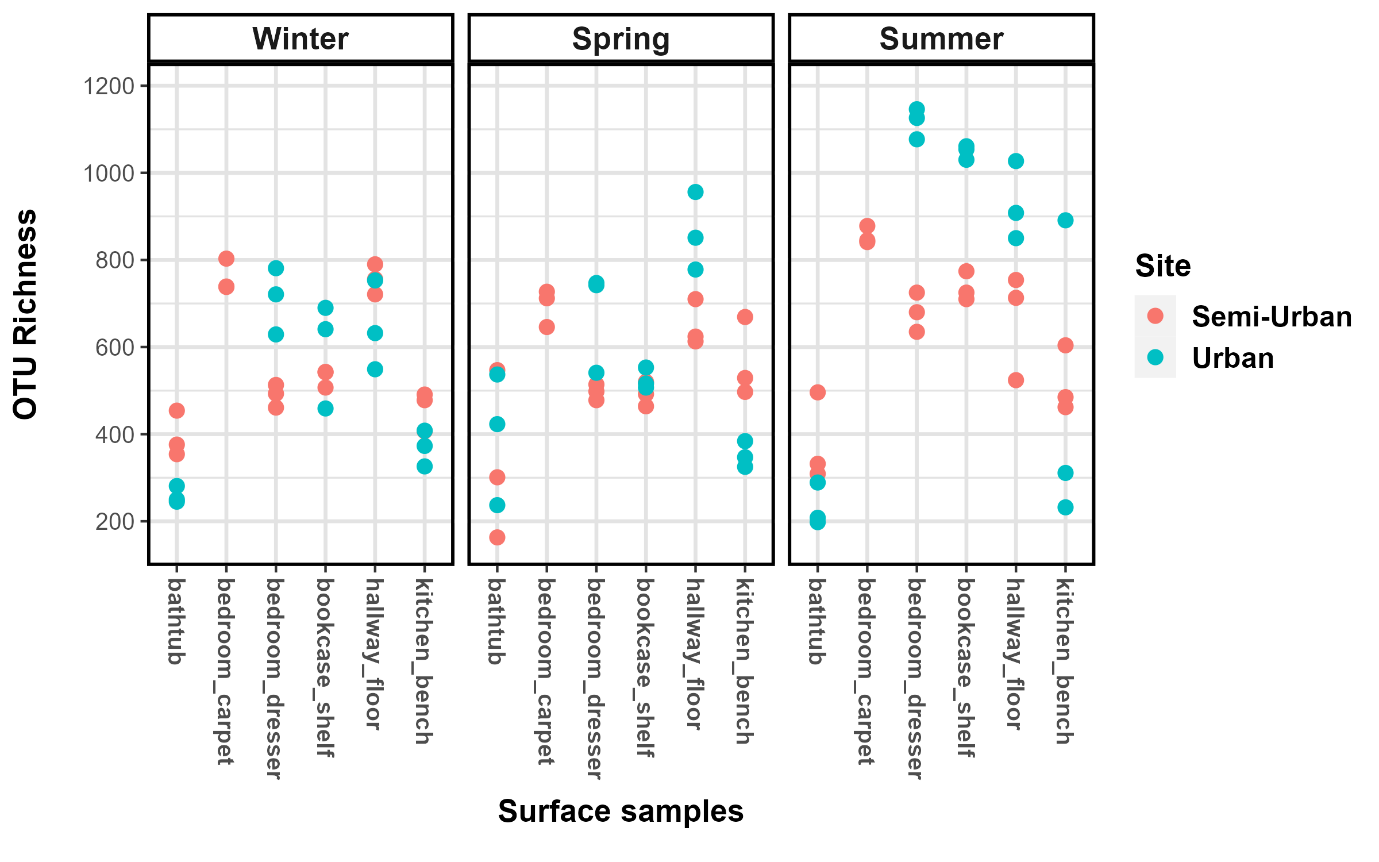


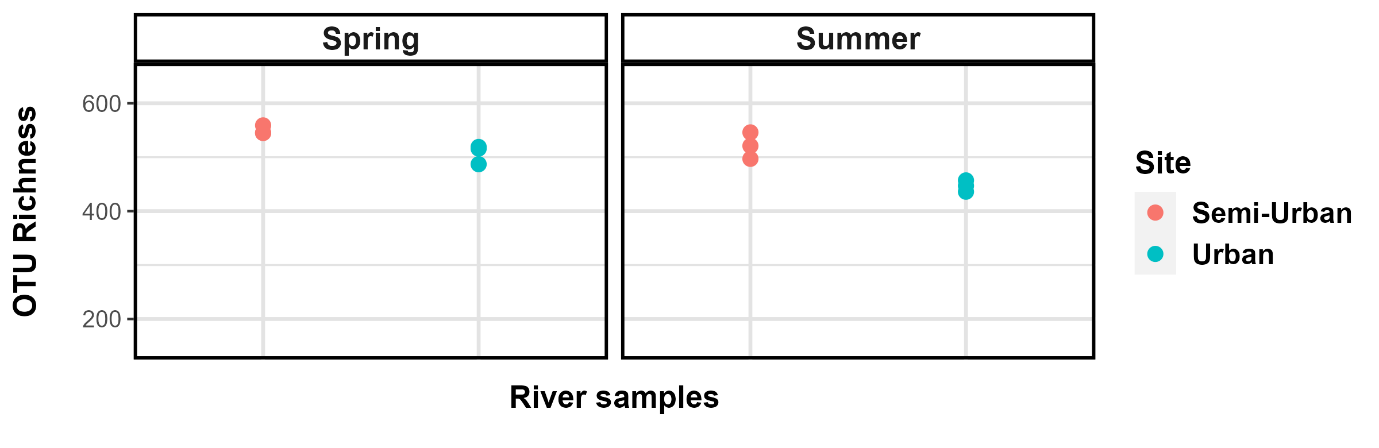
**Figure S9.** Numbers of observed bacterial OTUs for samples used for source tracking analysis: surface samples collected indoors (**top**) and water samples collected from the adjacent rivers (**bottom**), per each sampling site and season. River samples were not collected during winter.


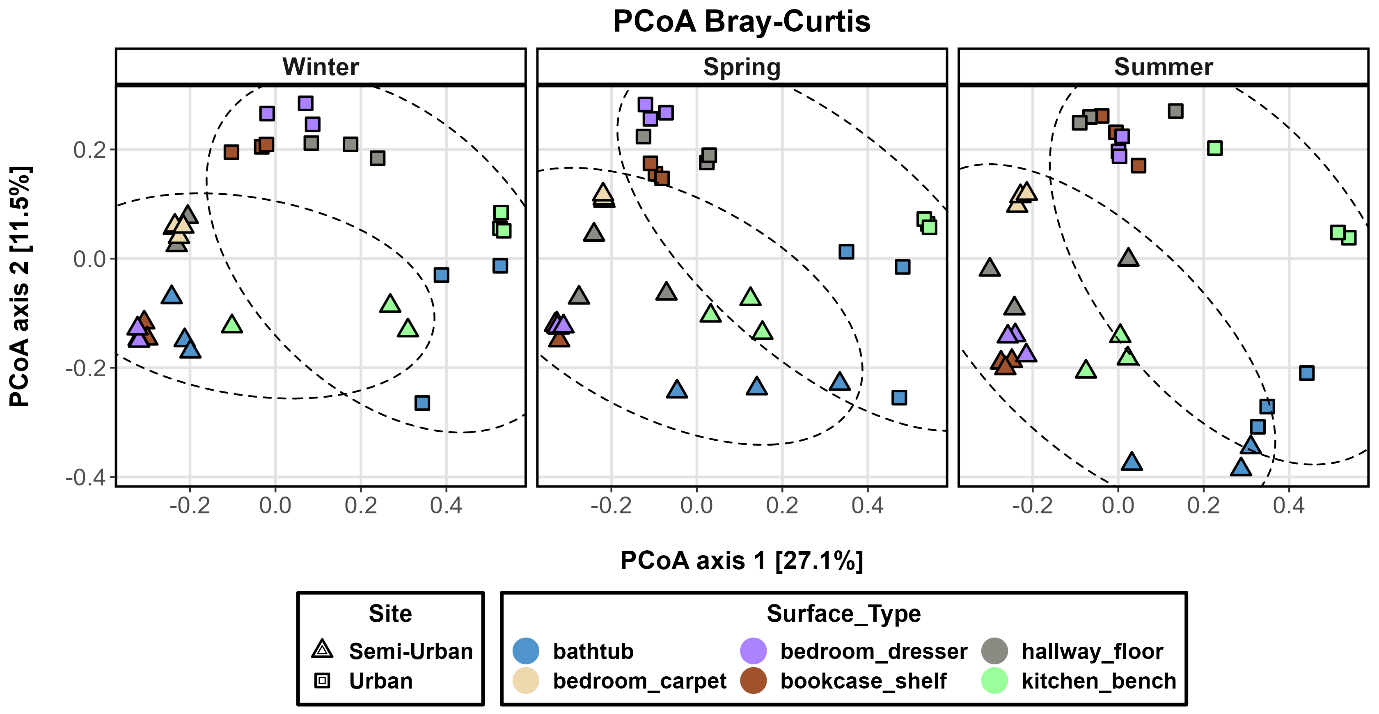


**Figure S10.** Principal coordinate analysis plots of bacterial beta diversity based on Bray-Curtis dissimilarity for interior surface samples collected indoors. Samples have different shapes based on site (urban/ semi-urban) and colours based on surface type. Sets of points corresponding to different sites are also annotated via ellipses.


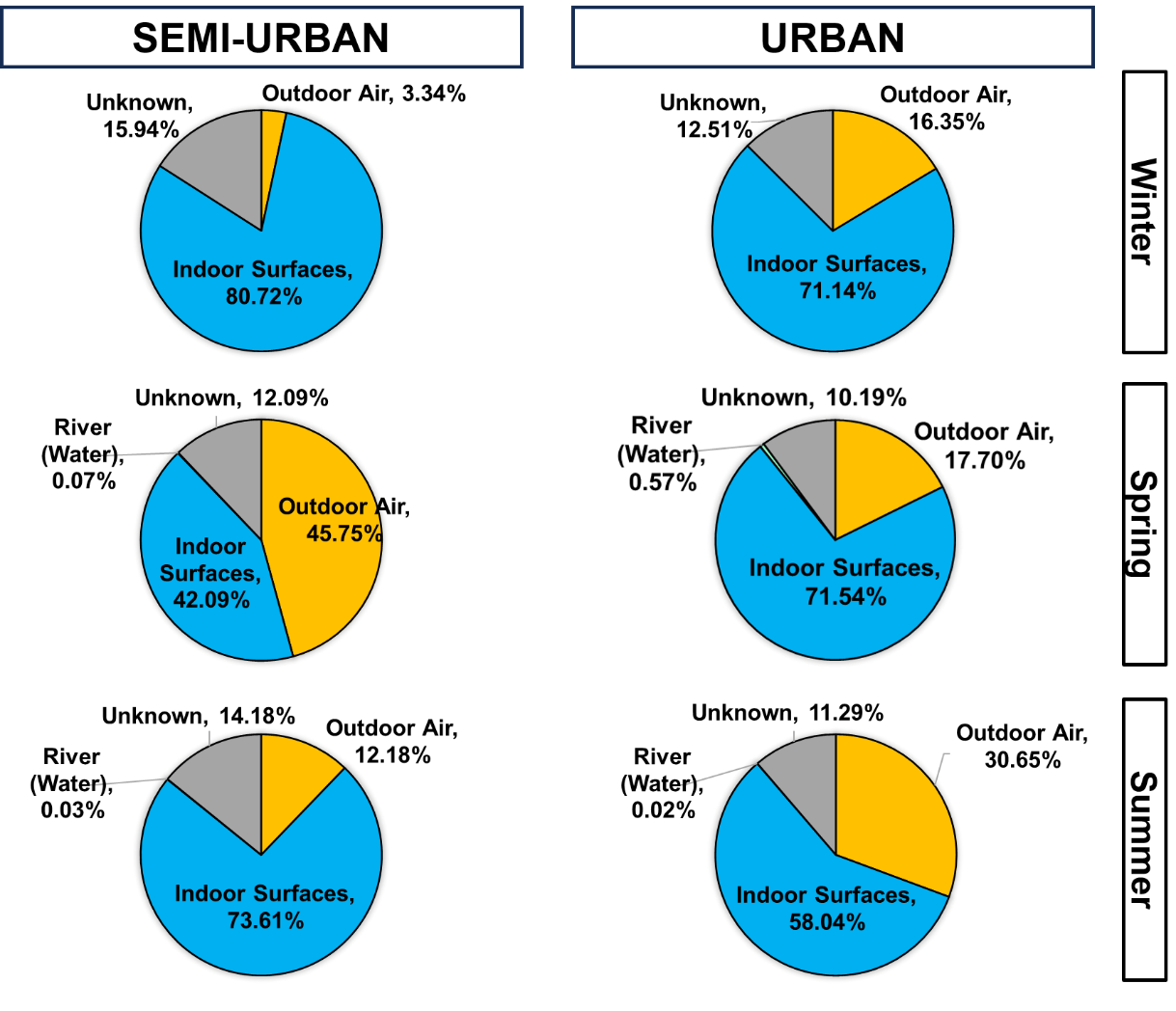


**Figure S11.** Fast Expectation-mAximization microbial Source Tracking (*FEAST*)-based average proportions of the contribution of various sources (outdoor air, interior surfaces, river water and unknown sources) to the overall indoor aerosol microbiome at both study sites over seasons.

**Supplementary Tables**

**Table S1.** Information on the characteristics of each household.

| **House**  **Characteristics** | **Urban flat**  **(London)** | **Semi-urban flat**  **(Colchester)** |
| --- | --- | --- |
| Building construction year | 2009 | 2011 |
| Building distance from closest river | ~20 m | ~10 m |
| Flat floor | 6 | 2 |
| No of bedrooms | 1 | 1 |
| Ventilation | Natural | Natural |
| HVAC | - | - |
| Dehumidifier | - | 1 |
| Floor type | Wood/tile | Wood/carpet |
| Carpeting | - | 1 (bedroom) |
| Stove type | Electric | Electric |
| Water damage/ visible mold issues | - | - |
| Occupants | 2 | 2 |
| Female:male | 1 | 1 |
| Smokers | 1 | 1 |
| Pets | - | - |
| Houseplants | 2 | - |

**Table S2.** Air sampling dates per each residential site and season.

| **Season**  **House** | | **Winter** | **Spring** | **Summer** |
| --- | --- | --- | --- | --- |
| **Urban**  **(London)** | **DAY 1** | 29.02.2016 | 11.05.2016 | 08.08.2016 |
|  | **DAY 2** | 02.03.2016 | 13.05.2016 | 11.08.2016 |
|  | **DAY 3** | 04.03.2016 | 16.05.2016 | 16.08.2016 |
| **Semi-urban**  **(Colchester)** | **DAY 1** | 22.02.2016 | 24.05.2016 | 22.08.2016 |
|  | **DAY 2** | 24.02.2016 | 27.05.2016 | 25.08.2016 |
|  | **DAY 3** | 26.02.2016 | 31.05.2016 | 30.08.2016 |

**Table S3*.*** List of samples collected from both residential sites.

| **Sampling method** | **Collection substrate** | **Flow  rate** | **Sampling environment** | **Duration** | **Replication** |
| --- | --- | --- | --- | --- | --- |
| 7-stage  May impactor | Glass slides | 20 LPM | Indoors  & Outdoors | Whole day (12 hours) | 3 days |
| Settled dust  sampling | Nylon swabs | n/a | Indoors  (six points) | n/a | 3 replicates |
| River water sampling* | 50 ml conical tubes | n/a | Outdoors | n/a | 3 replicates |

*River samples were only collected during spring and summer.

**Table S4*.*** Environmental parameters (mean ± standard deviations) measured.

|  | **OUTDOORS** | | | | | | | | **INDOORS** | | | |
| --- | --- | --- | --- | --- | --- | --- | --- | --- | --- | --- | --- | --- |
| Site | **London** | | | | **Colchester** | | | | **London** | | **Colchester** | |
|  | **Temp.**  **(°C)** | ***RH %** | **Wind**  **speed**  **(kph)** | **Precip. accum.**  **(mm)** | **Temp.**  **(°C)** | ***RH %** | **Wind**  **speed**  **(kph)** | **Precip. accum.**  **(mm)** | **Temp.**  **(°C)** | ***RH %** | **Temp.**  **(°C)** | ***RH %** |
| **WINTER** | | | | | | | | | | | | |
| **DAY 1** | 6.6  ± 1.7 | 69%  ± 8% | 4.0  ± 2.4 | -- | 6.8  ± 1.2 | 69%  ± 9% | 3.5  ± 2.7 | 1.8 | 21.5  ± 0.5 | 54%  ± 4% | 21.2  ± 1.2 | 49%  ± 3% |
| **DAY 2** | 5.7  ± 0.5 | 80%  ± 6% | 13.1  ± 4.5 | 3.0 | 5.8  ± 5.2 | 58%  ± 20% | 1.1  ± 1.1 | -- | 20.9  ± 0.5 | 52%  ± 3% | 21.2  ± 0.4 | 52%  ± 3% |
| **DAY 3** | 5.8  ± 2.4 | 69%  ± 11% | 6.9  ± 4.7 | 2.0 | 5.0  ± 3.3 | 59%  ± 10% | 3.1  ± 2.6 | -- | 22.7  ± 0.5 | 48%  ±4% | 21.7  ± 1.0 | 51%  ± 3% |
| **SPRING** | | | | | | | | | | | | |
| **DAY 1** | 17.2  ± 1.4 | 96%  ± 3% | 2.1  ± 1.2 | 8.1 | 16.1  ± 2.9 | 59%  ± 10% | 7.6  ± 1.6 | -- | 26.4  ± 0.6 | 55%  ± 3% | 25.2  ± 0.5 | 47%  ± 6% |
| **DAY 2** | 14.5  ± 2.9 | 78%  ± 6% | 11.0  ± 2.2 | -- | 19.8  ± 3.8 | 61%  ± 11% | 5.0  ± 1.6 | -- | 27.6  ± 0.6 | 44%  ± 2% | 25.1  ± 1.0 | 48%  ± 1% |
| **DAY 3** | 16.1  ± 2.8 | 63%  ± 14% | 4.2  ± 2.4 | -- | 12.0  ± 0.7 | 95%  ± 3% | 11.4  ± 2.8 | 36.8 | 27.0  ± 0.4 | 39%  ± 3% | 24.4  ± 0.5 | 55%  ± 2% |
| **SUMMER** | | | | | | | | | | | | |
| **DAY 1** | 19.2  ± 1.7 | 56%  ± 6% | 8.2  ± 3.9 | -- | 23.5  ± 3.0 | 62%  ± 10% | 7.0  ± 2.3 | 1.8 | 28.9  ± 1.0 | 33%  ±4% | 27.3  ± 1.6 | 48%  ± 3% |
| **DAY 2** | 20.4  ± 2.3 | 71%  ± 7% | 9.0  ± 3.1 | -- | 28.1  ± 4.2 | 57%  ± 13% | 2.8  ± 0.7 | -- | 27.9  ± 0.3 | 40%  ± 3 % | 28.9  ± 2.2 | 53%  ± 5% |
| **DAY 3** | 20.1  ± 2.7 | 71%  ± 12% | 4.9  ± 1.2 | -- | 24.7  ± 4.4 | 52%  ± 17% | 5.2  ± 2.2 | -- | 28.0  ± 0.9 | 40%  ± 2 % | 29.3  ± 3.5 | 43%  ± 6% |

*RH: Relative Humidity

**Table S5.** Indicator airborne bacterial genera of indoor and outdoor environments at the semi-urban site, based on the Indicator Value (stat) index (minimum positive predictive value A: 0.9 and minimum sensitivity B: 0.9).

| **Semi-urban (all seasons)** | | | | | | | | | |
| --- | --- | --- | --- | --- | --- | --- | --- | --- | --- |
| **Group Indoor: #sps. 1** | | | | | **Group Outdoor: #sps. 10** | | | | |
|  | **A** | **B** | **stat** | ***p*-value** |  | **A** | **B** | **stat** | ***p*-value** |
| *Anaerococcus* | 0.9317 | 0.9524 | 0.942 | 1.00E-04 | *Nocardioides* | 0.915 | 1 | 0.957 | 1.00E-04 |
|  |  |  |  |  | *Chryseobacterium* | 0.9059 | 1 | 0.952 | 1.00E-04 |
|  |  |  |  |  | *Geodermatophilus* | 0.9023 | 1 | 0.95 | 1.00E-04 |
|  |  |  |  |  | *Lysobacter* | 0.9337 | 0.9524 | 0.943 | 1.00E-04 |
|  |  |  |  |  | *Novosphingobium* | 0.9317 | 0.9524 | 0.942 | 1.00E-04 |
|  |  |  |  |  | *Flavobacterium* | 0.9279 | 0.9524 | 0.94 | 1.00E-04 |
|  |  |  |  |  | *Blastococcus* | 0.9118 | 0.9524 | 0.932 | 1.00E-04 |
|  |  |  |  |  | *Hymenobacter* | 0.9034 | 0.9524 | 0.928 | 1.00E-04 |
|  |  |  |  |  | *Rhodococcus* | 0.9194 | 0.9048 | 0.912 | 1.00E-04 |
|  |  |  |  |  | *Pedobacter* | 0.912 | 0.9048 | 0.908 | 9.00E-04 |
| **Winter** | | | | | | | | | |
| **Group Indoor: #sps. 2** | | | | | **Group Outdoor: #sps. 21** | | | | |
|  | **A** | **B** | **stat** | ***p*-value** |  | **A** | **B** | **stat** | ***p*-value** |
| *Anaerococcus* | 0.9831 | 1 | 0.992 | 0.0005 | *Erysipelothrix* | 1 | 1 | 1 | 0.0005 |
| *Propionibacterium* | 0.9558 | 1 | 0.978 | 0.0016 | *Paraperlucidibaca* | 1 | 1 | 1 | 0.0005 |
|  |  |  |  |  | *Sphingorhabdus* | 1 | 1 | 1 | 0.0005 |
|  |  |  |  |  | *Yaniella* | 1 | 1 | 1 | 0.0005 |
|  |  |  |  |  | *Hydrogenophaga* | 0.9954 | 1 | 0.998 | 0.0005 |
|  |  |  |  |  | *Jeotgalicoccus* | 0.9953 | 1 | 0.998 | 0.0005 |
|  |  |  |  |  | *Thermomonas* | 0.993 | 1 | 0.996 | 0.0005 |
|  |  |  |  |  | *Bacteroides* | 0.9811 | 1 | 0.99 | 0.0011 |
|  |  |  |  |  | *Altererythrobacter* | 0.9805 | 1 | 0.99 | 0.0012 |
|  |  |  |  |  | *Chryseobacterium* | 0.9787 | 1 | 0.989 | 0.0005 |
|  |  |  |  |  | *Novosphingobium* | 0.9768 | 1 | 0.988 | 0.0014 |
|  |  |  |  |  | *Blastococcus* | 0.9717 | 1 | 0.986 | 0.0005 |
|  |  |  |  |  | *Nocardioides* | 0.9682 | 1 | 0.984 | 0.0005 |
|  |  |  |  |  | *Marmoricola* | 0.9647 | 1 | 0.982 | 0.0005 |
|  |  |  |  |  | *Psychrobacter* | 0.9628 | 1 | 0.981 | 0.0005 |
|  |  |  |  |  | *Clostridium_sensu_stricto* | 0.958 | 1 | 0.979 | 0.0018 |
|  |  |  |  |  | *Saccharopolyspora* | 0.9518 | 1 | 0.976 | 0.0466 |
|  |  |  |  |  | *Flavobacterium* | 0.9502 | 1 | 0.975 | 0.0005 |
|  |  |  |  |  | *Arthrobacter* | 0.9358 | 1 | 0.967 | 0.0005 |
|  |  |  |  |  | *Curtobacterium* | 0.917 | 1 | 0.958 | 0.0016 |
|  |  |  |  |  | *Rubellimicrobium* | 0.9108 | 1 | 0.954 | 0.0113 |
| **Spring** | | | | | | | | | |
| **Group Indoor: #sps. 6** | | | | | **Group Outdoor: #sps. 31** | | | | |
|  | **A** | **B** | **stat** | ***p*-value** |  | **A** | **B** | **stat** | ***p*-value** |
| *Peptoniphilus* | 1 | 1 | 1 | 0.001 | *Chryseomicrobium* | 1 | 1 | 1 | 0.001 |
| *Anaerococcus* | 0.9945 | 1 | 0.997 | 0.001 | *Paenibacillus* | 0.9946 | 1 | 0.997 | 0.001 |
| *Propionibacterium* | 0.988 | 1 | 0.994 | 0.001 | *Citricoccus* | 0.9929 | 1 | 0.996 | 0.001 |
| *Finegoldia* | 0.9835 | 1 | 0.992 | 0.0016 | *Thermomonas* | 0.9916 | 1 | 0.996 | 0.001 |
| *Haematobacter* | 0.9581 | 1 | 0.979 | 0.001 | *Ilumatobacter* | 0.9891 | 1 | 0.995 | 0.001 |
| *Thalassolituus* | 0.9026 | 1 | 0.95 | 0.0066 | *Lysobacter* | 0.988 | 1 | 0.994 | 0.001 |
|  |  |  |  |  | *Glaciimonas* | 0.9878 | 1 | 0.994 | 0.001 |
|  |  |  |  |  | *Saccharopolyspora* | 0.9839 | 1 | 0.992 | 0.001 |
|  |  |  |  |  | *Thermoactinomyces* | 0.9823 | 1 | 0.991 | 0.001 |
|  |  |  |  |  | *Roseococcus* | 0.9814 | 1 | 0.991 | 0.0026 |
|  |  |  |  |  | *Rufibacter* | 0.9803 | 1 | 0.99 | 0.001 |
|  |  |  |  |  | *Flavisolibacter* | 0.9781 | 1 | 0.989 | 0.001 |
|  |  |  |  |  | *Solirubrobacter* | 0.9727 | 1 | 0.986 | 0.001 |
|  |  |  |  |  | *Nocardioides* | 0.9681 | 1 | 0.984 | 0.001 |
|  |  |  |  |  | *Noviherbaspirillum* | 0.9642 | 1 | 0.982 | 0.001 |
|  |  |  |  |  | *Blastococcus* | 0.9605 | 1 | 0.98 | 0.001 |
|  |  |  |  |  | *Marmoricola* | 0.9598 | 1 | 0.98 | 0.001 |
|  |  |  |  |  | *Cellulomonas* | 0.959 | 1 | 0.979 | 0.0017 |
|  |  |  |  |  | *Herminiimonas* | 0.9573 | 1 | 0.978 | 0.001 |
|  |  |  |  |  | *Hymenobacter* | 0.9559 | 1 | 0.978 | 0.001 |
|  |  |  |  |  | *Bacillus* | 0.955 | 1 | 0.977 | 0.001 |
|  |  |  |  |  | *Acidovorax* | 0.9456 | 1 | 0.972 | 0.0024 |
|  |  |  |  |  | *Rhodococcus* | 0.9442 | 1 | 0.972 | 0.001 |
|  |  |  |  |  | *Streptomyces* | 0.9398 | 1 | 0.969 | 0.001 |
|  |  |  |  |  | *Arthrobacter* | 0.9356 | 1 | 0.967 | 0.001 |
|  |  |  |  |  | *Novosphingobium* | 0.9326 | 1 | 0.966 | 0.001 |
|  |  |  |  |  | *Rubellimicrobium* | 0.9323 | 1 | 0.966 | 0.001 |
|  |  |  |  |  | *Clostridium_sensu_stricto* | 0.9302 | 1 | 0.964 | 0.0017 |
|  |  |  |  |  | *Skermanella* | 0.9282 | 1 | 0.963 | 0.0022 |
|  |  |  |  |  | *Geodermatophilus* | 0.9208 | 1 | 0.96 | 0.0021 |
|  |  |  |  |  | *Chryseobacterium* | 0.9131 | 1 | 0.956 | 0.0031 |
| **Summer** | | | | | | | | | |
| **Group Indoor: #sps. 4** | | | | | **Group Outdoor: #sps. 3** | | | | |
|  | **A** | **B** | **stat** | ***p*-value** |  | **A** | **B** | **stat** | ***p*-value** |
| *Peptoniphilus* | 1 | 1 | 1 | 8.00E-04 | *Flavobacterium* | 0.9362 | 1 | 0.968 | 0.0008 |
| *Haematobacter* | 0.9883 | 1 | 0.994 | 8.00E-04 | *Microbacterium* | 0.9339 | 1 | 0.966 | 0.0027 |
| *Streptococcus* | 0.9576 | 1 | 0.979 | 8.00E-04 | *Geodermatophilus* | 0.9234 | 1 | 0.961 | 0.0008 |
| *Brevibacterium* | 0.9161 | 1 | 0.957 | 8.00E-04 |  |  |  |  |  |

**Table S6.** Indicator airborne bacterial genera of indoor and outdoor environments at the urban site, based on the Indicator Value (stat) index (minimum positive predictive value A: 0.9 and minimum sensitivity B: 0.9).

| **Urban (all seasons)** | | | | | | | | | |
| --- | --- | --- | --- | --- | --- | --- | --- | --- | --- |
| **Group Indoor: #sps. 2** | | | | | **Group Outdoor: #sps. 5** | | | | |
|  | **A** | **B** | **stat** | ***p*-value** |  | **A** | **B** | **stat** | ***p*-value** |
| *Propionibacterium* | 0.9578 | 1 | 0.979 | 1.00E-04 | *Planococcus* | 0.9479 | 0.9524 | 0.95 | 1.00E-04 |
| *Finegoldia* | 0.9303 | 0.9048 | 0.917 | 1.00E-04 | *Marmoricola* | 0.9411 | 0.9524 | 0.947 | 1.00E-04 |
|  |  |  |  |  | *Herminiimonas* | 0.9371 | 0.9524 | 0.945 | 1.00E-04 |
|  |  |  |  |  | *Altererythrobacter* | 0.933 | 0.9524 | 0.943 | 1.00E-04 |
|  |  |  |  |  | *Modestobacter* | 0.9562 | 0.9048 | 0.93 | 1.00E-04 |
| **Winter** | | | | | | | | | |
| **Group Indoor: #sps. 14** | | | | | **Group Outdoor: #sps. 25** | | | | |
|  | **A** | **B** | **stat** | ***p*-value** |  | **A** | **B** | **stat** | ***p*-value** |
| *Anaerostipes* | 1 | 1 | 1 | 0.0004 | *Cellulomonas* | 1 | 1 | 1 | 0.0004 |
| *Clostridium_XVIII* | 1 | 1 | 1 | 0.0004 | *Spirosoma* | 0.9927 | 1 | 0.996 | 0.0004 |
| *Ezakiella* | 0.9883 | 1 | 0.994 | 0.0004 | *Lysobacter* | 0.9922 | 1 | 0.996 | 0.0004 |
| *Collinsella* | 0.9812 | 1 | 0.991 | 0.0004 | *Gillisia* | 0.9902 | 1 | 0.995 | 0.0004 |
| *Neisseria* | 0.9599 | 1 | 0.98 | 0.0011 | *Herminiimonas* | 0.9894 | 1 | 0.995 | 0.0004 |
| *Ruminococcus2* | 0.945 | 1 | 0.972 | 0.0004 | *Altererythrobacter* | 0.9866 | 1 | 0.993 | 0.0004 |
| *Propionibacterium* | 0.9437 | 1 | 0.971 | 0.0004 | *Gaiella* | 0.982 | 1 | 0.991 | 0.0004 |
| *Finegoldia* | 0.9416 | 1 | 0.97 | 0.0004 | *Friedmanniella* | 0.9808 | 1 | 0.99 | 0.0004 |
| *Ruminococcus* | 0.9351 | 1 | 0.967 | 0.0004 | *Trichococcus* | 0.9778 | 1 | 0.989 | 0.0004 |
| *Peptoniphilus* | 0.9327 | 1 | 0.966 | 0.0004 | *Psychrobacter* | 0.9755 | 1 | 0.988 | 0.0004 |
| *Actinotignum* | 0.9326 | 1 | 0.966 | 0.0004 | *Hymenobacter* | 0.9753 | 1 | 0.988 | 0.0004 |
| *Anaerococcus* | 0.9324 | 1 | 0.966 | 0.0008 | *Variovorax* | 0.973 | 1 | 0.986 | 0.0017 |
| *Haemophilus* | 0.9239 | 1 | 0.961 | 0.0051 | *Curtobacterium* | 0.972 | 1 | 0.986 | 0.0004 |
| *Porphyromonas* | 0.9155 | 1 | 0.957 | 0.0013 | *Marmoricola* | 0.9681 | 1 | 0.984 | 0.001 |
|  |  |  |  |  | *Geodermatophilus* | 0.9668 | 1 | 0.983 | 0.0004 |
|  |  |  |  |  | *Rhodococcus* | 0.9634 | 1 | 0.982 | 0.001 |
|  |  |  |  |  | *Nocardioides* | 0.9619 | 1 | 0.981 | 0.0004 |
|  |  |  |  |  | *Blastococcus* | 0.959 | 1 | 0.979 | 0.0004 |
|  |  |  |  |  | *Thermomonas* | 0.9457 | 1 | 0.972 | 0.0013 |
|  |  |  |  |  | *Truepera* | 0.9431 | 1 | 0.971 | 0.0022 |
|  |  |  |  |  | *Massilia* | 0.9241 | 1 | 0.961 | 0.0004 |
|  |  |  |  |  | *Novosphingobium* | 0.9238 | 1 | 0.961 | 0.0013 |
|  |  |  |  |  | *Rathayibacter* | 0.9202 | 1 | 0.959 | 0.0004 |
|  |  |  |  |  | *Mycobacterium* | 0.9201 | 1 | 0.959 | 0.0017 |
|  |  |  |  |  | *Erythrobacter* | 0.9077 | 1 | 0.953 | 0.0105 |
| **Spring** | | | | | | | | | |
| **Group Indoor: #sps. 3** | | | | | **Group Outdoor: #sps. 11** | | | | |
|  | **A** | **B** | **stat** | ***p*-value** |  | **A** | **B** | **stat** | ***p*-value** |
| *Propionibacterium* | 0.989 | 1 | 0.994 | 0.0008 | *Planifilum* | 0.9937 | 1 | 0.997 | 0.0008 |
| *Actinomyces* | 0.972 | 1 | 0.986 | 0.0046 | *Planococcus* | 0.9783 | 1 | 0.989 | 0.0008 |
| *Ruminococcus* | 0.9346 | 1 | 0.967 | 0.0012 | *Modestobacter* | 0.9722 | 1 | 0.986 | 0.0029 |
|  |  |  |  |  | *Psychrobacter* | 0.9584 | 1 | 0.979 | 0.0017 |
|  |  |  |  |  | *Rubellimicrobium* | 0.9321 | 1 | 0.965 | 0.0008 |
|  |  |  |  |  | *Rathayibacter* | 0.9307 | 1 | 0.965 | 0.0025 |
|  |  |  |  |  | *Curtobacterium* | 0.9294 | 1 | 0.964 | 0.0008 |
|  |  |  |  |  | *Pedobacter* | 0.9251 | 1 | 0.962 | 0.0013 |
|  |  |  |  |  | *Clostridium_sensu_stricto* | 0.9187 | 1 | 0.958 | 0.0012 |
|  |  |  |  |  | *Exiguobacterium* | 0.9085 | 1 | 0.953 | 0.0053 |
|  |  |  |  |  | *Citricoccus* | 0.9062 | 1 | 0.952 | 0.0008 |
| **Summer** | | | | | | | | | |
| **Group Indoor: #sps. 6** | | | | | **Group Outdoor: #sps. 1** | | | | |
|  | **A** | **B** | **stat** | ***p*-value** |  | **A** | **B** | **stat** | ***p*-value** |
| *Haematobacter* | 1 | 1 | 1 | 5.00E-04 | *Planococcus* | 0.9427 | 1 | 0.971 | 0.0025 |
| *Finegoldia* | 0.9762 | 1 | 0.988 | 5.00E-04 |  |  |  |  |  |
| *Anaerococcus* | 0.9603 | 1 | 0.98 | 5.00E-04 |  |  |  |  |  |
| *Propionibacterium* | 0.948 | 1 | 0.974 | 5.00E-04 |  |  |  |  |  |
| *Pseudoxanthomonas* | 0.9477 | 1 | 0.973 | 5.00E-04 |  |  |  |  |  |
| *Peptoniphilus* | 0.9263 | 1 | 0.962 | 5.00E-04 |  |  |  |  |  |

**Table S7.** Indicator airborne bacterial genera of the urban and semi-urban* sites for the indoor environment, based on the Indicator Value (stat) index (minimum positive predictive value A: 0.7 and minimum sensitivity B: 0.7).

| **INDOOR (all seasons)** | | | | |
| --- | --- | --- | --- | --- |
| **Group Urban: #sps. 17** | | | | |
|  | **A** | **B** | **stat** | ***p*-value** |
| *Stenotrophomonas* | 0.9402 | 0.9524 | 0.946 | 0.0001 |
| *Blautia* | 0.921 | 0.9524 | 0.937 | 0.0001 |
| *Aerococcus* | 0.9548 | 0.9048 | 0.929 | 0.0001 |
| *Sphingobium* | 0.8895 | 0.9524 | 0.92 | 0.0001 |
| *Roseburia* | 0.9291 | 0.9048 | 0.917 | 0.0001 |
| *Rheinheimera* | 0.9574 | 0.8571 | 0.906 | 0.0001 |
| *Rhodococcus* | 0.902 | 0.8571 | 0.879 | 0.0036 |
| *Ruminococcus2* | 0.8892 | 0.8571 | 0.873 | 0.0003 |
| *Clostridium_XVIII* | 0.9842 | 0.7619 | 0.866 | 0.0001 |
| *Bacteroides* | 0.8738 | 0.8571 | 0.865 | 0.0005 |
| *Ruminococcus* | 0.7919 | 0.9048 | 0.846 | 0.0004 |
| *Tepidimicrobium* | 0.8735 | 0.8095 | 0.841 | 0.0002 |
| *Skermanella* | 0.8443 | 0.8095 | 0.827 | 0.0002 |
| *Collinsella* | 0.8173 | 0.8095 | 0.813 | 0.0004 |
| *Clostridium_XlVa* | 0.8013 | 0.8095 | 0.805 | 0.0033 |
| *Deinococcus* | 0.7904 | 0.8095 | 0.8 | 0.002 |
| *Actinotignum* | 0.714 | 0.7143 | 0.714 | 0.0039 |

**Table S8.** Indicator airborne bacterial genera of the urban and semi-urban* sites for the outdoor environment, based on the Indicator Value (stat) index (minimum positive predictive value A: 0.7 and minimum sensitivity B: 0.7).

| **OUTDOOR (all seasons)** | | | | |
| --- | --- | --- | --- | --- |
| **Group Urban: #sps. 3** | | | | |
|  | **A** | **B** | **stat** | ***p*-value** |
| *Actinoallomurus* | 0.8286 | 0.8571 | 0.843 | 0.001 |
| *Comamonas* | 0.8499 | 0.7143 | 0.779 | 0.0061 |
| *Lactococcus* | 0.757 | 0.7143 | 0.735 | 0.0133 |

*No results for the specified threshold.

**Table S9.** Indicator airborne bacterial genera of particles < 2 μm and > 2 μm for the indoor and outdoor environment (both sites), based on the Indicator Value (stat) index (minimum positive predictive value A: 0.7 and minimum sensitivity B: 0.7).

| **INDOOR (both sites)** | | | | | | | | | |
| --- | --- | --- | --- | --- | --- | --- | --- | --- | --- |
| **Group < 2 μm: #sps. 4** | | | | | **Group > 2 μm: #sps. 1** | | | | |
|  | **A** | **B** | **stat** | ***p*-value** |  | **A** | **B** | **stat** | ***p*-value** |
| *Streptomyces* | 0.9066 | 1 | 0.952 | 0.0001 | *Facklamia* | 0.8256 | 0.9583 | 0.89 | 0.0002 |
| *Saccharopolyspora* | 0.8429 | 0.8889 | 0.866 | 0.0002 |  |  |  |  |  |
| *Thermoactinomyces* | 0.9327 | 0.7778 | 0.852 | 0.0006 |  |  |  |  |  |
| *Pseudonocardia* | 0.8927 | 0.7222 | 0.803 | 0.0014 |  |  |  |  |  |
| **OUTDOOR (both sites)** | | | | | | | | | |
| **Group < 2 μm: #sps. 3** | | | | | **Group > 2 μm: #sps. 5** | | | | |
|  | **A** | **B** | **stat** | ***p*-value** |  | **A** | **B** | **stat** | ***p*-value** |
| *Nocardiopsis* | 0.9801 | 0.8333 | 0.904 | 0.0001 | *Nakamurella* | 0.8093 | 1 | 0.9 | 0.0001 |
| *Saccharomonospora* | 0.7904 | 0.9444 | 0.864 | 0.0002 | *Variovorax* | 0.8218 | 0.9583 | 0.887 | 0.0001 |
| *Nonomuraea* | 0.855 | 0.8333 | 0.844 | 0.0006 | *Belnapia* | 0.8561 | 0.7917 | 0.823 | 0.0003 |
|  |  |  |  |  | *Caldimonas* | 0.8194 | 0.7917 | 0.805 | 0.0021 |
|  |  |  |  |  | *Gillisia* | 0.8142 | 0.75 | 0.781 | 0.0233 |

**Table S10.** Beta diversity statistics based on Bray-Curtis dissimilarities
(9999 permutations). Bolded values indicate statistical significance.

| **Subset of Samples (Impactor)** | **Factor** | **Df** | **PERMANOVA** | | | **PERMDISP** | |
| --- | --- | --- | --- | --- | --- | --- | --- |
|  |  |  | **R^2^** | **Pseudo-*F*** | ***p*-value** | ***F*** | ***p*-value** |
| **Both sites (IN & OUT)** | **Site** | 1 | 0.04993 | 4.3091 | **4.00E-04** | 9.9091 | **0.0021** |
| **< 2 µm (fine)** | Site | 1 | 0.08031 | 2.9691 | **0.0031** | 6.9698 | **0.0131** |
| **> 2 µm (coarse)** | Site | 1 | 0.05028 | 2.4351 | **0.0237** | 2.6933 | 0.1132 |
|  |  |  |  |  |  |  |  |
| **INDOOR (both sites)** | Site | 1 | 0.14111 | 6.5716 | **1.00E-04** | 2.3857 | 0.1296 |
| **INDOOR < 2 µm (fine)** | Site | 1 | 0.1908 | 3.7725 | **1.00E-04** | 3.0906 | 0.0958 |
| **INDOOR > 2 µm (coarse))** | Site | 1 | 0.1677 | 4.4328 | **1.00E-04** | 0.0026 | 0.957 |
|  |  |  |  |  |  |  |  |
| **OUTDOOR (both sites)** | Site | 1 | 0.05515 | 2.3346 | **0.0011** | 7.2396 | **0.0111** |
| **OUTDOOR < 2 µm (fine)** | Site | 1 | 0.0975 | 1.7286 | **0.006** | 7.9012 | **0.0131** |
| **OUTDOOR > 2 µm (coarse)** | Site | 1 | 0.0714 | 1.6916 | **0.038** | 4.4012 | **0.0494** |
|  |  |  |  |  |  |  |  |
| **Both sites (all seasons)** | **Site & Environment** | 3 | 0.28635 | 10.7 | **1.00E-04** | 18.169 | **1.00E-04** |
|  |  |  |  |  |  |  |  |
| **Both sites (all seasons)** | **Environment** | 1 | 0.21645 | 22.651 | **1.00E-04** | 33.04 | **1.00E-04** |
| **< 2 µm (fine)** | Environment | 1 | 0.19368 | 8.1669 | **1.00E-04** | 18.622 | **1.00E-04** |
| **> 2 µm (coarse)** | Environment | 1 | 0.29315 | 19.077 | **1.00E-04** | 16.815 | **1.00E-04** |
|  |  |  |  |  |  |  |  |
| **URBAN (all seasons)** | Environment | 1 | 0.25585 | 13.752 | **1.00E-04** | 25.206 | **1.00E-04** |
| **URBAN Winter** | Environment | 1 | 0.43916 | 9.3964 | **0.0011** | 52.071 | **1.00E-04** |
| **URBAN Spring** | Environment | 1 | 0.35835 | 6.7017 | **5.00E-04** | 5.182 | **0.0423** |
| **URBAN Summer** | Environment | 1 | 0.30305 | 5.2179 | **7.00E-04** | 0.002 | 0.9655 |
| **SEMI-URBAN (all seasons)** | Environment | 1 | 0.24322 | 12.855 | **1.00E-04** | 21.67 | **1.00E-04** |
| **SEMI-URBAN Winter** | Environment | 1 | 0.42431 | 8.8446 | **2.00E-04** | 75.032 | **1.00E-04** |
| **SEMI-URBAN Spring** | Environment | 1 | 0.3616 | 6.7968 | **0.0011** | 1.3667 | 0.2444 |
| **SEMI-URBAN Summer** | Environment | 1 | 0.34326 | 6.2721 | **0.0011** | 25.708 | **4.00E-04** |
|  |  |  |  |  |  |  |  |
| **Both sites (IN & OUT)** | **Season** | 2 | 0.12122 | 5.5864 | **1.00E-04** | 0.6786 | 0.515 |
| **< 2 µm (fine)** | Season | 2 | 0.15964 | 3.1343 | **2.00E-04** | 0.0029 | 0.9966 |
| **> 2 µm (coarse)** | Season | 2 | 0.13987 | 3.6588 | **3.00E-04** | 2.0268 | 0.1466 |
|  |  |  |  |  |  |  |  |
| **INDOOR (both sites)** | Season | 2 | 0.21715 | 5.4089 | **1.00E-04** | 7.7939 | **6.00E-04** |
| **< 2 µm (fine)** | Season | 2 | 0.33923 | 3.8504 | **2.00E-04** | 7.5528 | **0.0019** |
| **> 2 µm (coarse)** | Season | 2 | 0.24651 | 3.4352 | **1.00E-04** | 6.3818 | **0.0105** |
|  |  |  |  |  |  |  |  |
| **INDOOR URBAN** | Season | 2 | 0.35807 | 5.0202 | **1.00E-04** | 3.8883 | **0.0335** |
| **INDOOR URBAN  < 2 µm (fine)** | Season | 2 | 0.53601 | 3.4656 | **0.0024** | 1.5582 | 0.3088 |
| **INDOOR URBAN  > 2 µm (coarse)** | Season | 2 | 0.50663 | 4.6209 | **2.00E-04** | 1.0335 | 0.4036 |
|  |  |  |  |  |  |  |  |
| **INDOOR SEMI-URBAN** | Season | 2 | 0.44727 | 7.2828 | **1.00E-04** | 1.2622 | 0.3123 |
| **INDOOR SEMI-URBAN  < 2 µm (fine)** | Season | 2 | 0.76946 | 10.013 | **0.0033** | 0.9631 | 0.4359 |
| **INDOOR SEMI-URBAN  > 2 µm (coarse)** | Season | 2 | 0.45949 | 3.8255 | **2.00E-04** | 1.0487 | 0.3834 |
|  |  |  |  |  |  |  |  |
| **OUTDOOR (both sites)** | Season | 2 | 0.19024 | 4.5814 | **1.00E-04** | 2.1585 | 0.1301 |
| **OUTDOOR < 2 µm (fine)** | Season | 2 | 0.22663 | 2.1978 | **1.00E-04** | 0.2964 | 0.7517 |
| **OUTDOOR > 2 µm (coarse)** | Season | 2 | 0.28098 | 4.1033 | **1.00E-04** | 4.3388 | **0.025** |
|  |  |  |  |  |  |  |  |
| **OUTDOOR URBAN** | Season | 2 | 0.27266 | 3.3739 | **1.00E-04** | 3.1354 | 0.0677 |
| **OUTDOOR URBAN  < 2 µm (fine)** | Season | 2 | 0.37106 | 1.7699 | **0.0117** | 2.0555 | 0.2452 |
| **OUTDOOR URBAN  > 2 µm (coarse)** | Season | 2 | 0.4375 | 3.5 | **3.00E-04** | 5.4203 | **0.0196** |
|  |  |  |  |  |  |  |  |
| **OUTDOOR SEMI-URBAN** | Season | 2 | 0.26038 | 3.1685 | **1.00E-04** | 4.8142 | **0.0233** |
| **OUTDOOR SEMI-URBAN**  **< 2 µm (fine)** | Season | 2 | 0.41288 | 2.1096 | **0.0039** | 3.6035 | 0.1269 |
| **OUTDOOR SEMI-URBAN**  **> 2 µm (coarse)** | Season | 2 | 0.36865 | 2.6276 | **2.00E-04** | 12.215 | **0.0044** |
|  |  |  |  |  |  |  |  |
| **Both sites (all seasons)** | **Stage** | 6 | 0.0765 | 1.063 | 0.3202 | 0.11 | 0.9957 |
| **INDOOR (both sites)** | Stage | 6 | 0.18938 | 1.3628 | **0.0433** | 0.1567 | 0.9907 |
| **OUTDOOR (both sites)** | Stage | 6 | 0.15772 | 1.0923 | 0.1822 | 2.0287 | 0.0873 |
|  |  |  |  |  |  |  |  |
| **Both sites (all)** | **Size fraction** | 1 | 0.0403 | 3.4437 | **0.0012** | 0.0109 | 0.9138 |
| **URBAN (all)** | Size fraction | 1 | 0.06465 | 2.7646 | **0.0125** | 1.4981 | 0.2196 |
| **INDOOR URBAN** | Size fraction | 1 | 0.16594 | 3.7801 | **5.00E-04** | 3.9701 | 0.058 |
| **OUTDOOR URBAN** | Size fraction | 1 | 0.10787 | 2.2973 | **0.0066** | 2.7744 | 0.1101 |
| **SEMI-URBAN (all)** | Size fraction | 1 | 0.04506 | 1.8874 | **0.0472** | 0.4808 | 0.4958 |
| **INDOOR SEMI-URBAN** | Size fraction | 1 | 0.13161 | 2.8795 | **0.0202** | 0.6591 | 0.4439 |
| **OUTDOOR SEMI-URBAN** | Size fraction | 1 | 0.08003 | 1.6528 | **0.0195** | 3.3118 | 0.0895 |

**Table S11.** Beta diversity statistics based on Bray-Curtis dissimilarities
(9999 permutations). Bolded values indicate statistical significance*.*

| **Subset of Samples** | **Factor** | **Df** | **PERMANOVA** | | | **PERMDISP** | |
| --- | --- | --- | --- | --- | --- | --- | --- |
|  |  |  | **R^2^** | **Pseudo-*F*** | ***p*-value** | ***F*** | ***p*-value** |
| **Surface & Impactor** |  |  |  |  |  |  |  |
| **SEMI-URBAN Winter** | **Sample Type** | 1 | 0.13161 | 3.4858 | **0.0013** | 2.0663 | 0.1682 |
| **SEMI-URBAN Spring** | Sample Type | 1 | 0.22523 | 6.6862 | **2.00E-04** | 1.238 | 0.2724 |
| **SEMI-URBAN Summer** | Sample Type | 1 | 0.14487 | 3.8965 | **1.00E-04** | 2.2598 | 0.147 |
| **URBAN Winter** | Sample Type | 1 | 0.2182 | 5.5818 | **9.00E-04** | 13.202 | **0.0016** |
| **URBAN Spring** | Sample Type | 1 | 0.15854 | 3.7681 | **0.005** | 9.2917 | **0.0063** |
| **URBAN Summer** | Sample Type | 1 | 0.13262 | 3.0581 | **0.0078** | 3.459 | 0.088 |
|  |  |  |  |  |  |  |  |
| **Surface (both sites)** | **Site** | 1 | 0.15065 | 17.205 | **1.00E-04** | 7.9312 | **0.005** |
| **Surface Winter** | Site | 1 | 0.24864 | 10.258 | **1.00E-04** | 3.2154 | 0.0811 |
| **Surface Spring** | Site | 1 | 0.16179 | 5.9835 | **1.00E-04** | 0.9902 | 0.3165 |
| **Surface Summer** | Site | 1 | 0.17348 | 6.5066 | **1.00E-04** | 0.4311 | 0.5149 |
|  |  |  |  |  |  |  |  |
| **Surface SEMI-URBAN** | **Season** | 2 | 0.08814 | 2.4648 | **0.0012** | 0.9244 | 0.405 |
| **Surface URBAN** | Season | 2 | 0.1192 | 2.8419 | **0.0018** | 0.1194 | 0.8867 |

**Table S12.** Beta diversity statistics based on Bray-Curtis dissimilarities
(9999 permutations). Bolded values indicate statistical significance*.*

| **PERMANOVA (pairwise)  Subset of Samples: Surface, Site: URBAN, Factor: Surface Type** | | | | | | | | |
| --- | --- | --- | --- | --- | --- | --- | --- | --- |
| # | **pairs** | | | **Df** | **F. Model** | **R^2^** | ***p*-value** | ***p-*adjusted** |
| 1 | Bookcase shelf | vs | Bedroom dresser | 1 | 1.35661 | 0.07816 | 0.1635 | 1 |
| 2 | Bookcase shelf | vs | Bathtub | 1 | 9.39694 | 0.37 | **0.0001** | **0.001** |
| 3 | Bookcase shelf | vs | Kitchen bench | 1 | 17.30855 | 0.51964 | **0.0001** | **0.001** |
| 4 | Bookcase shelf | vs | Hallway floor | 1 | 2.60816 | 0.14016 | **0.0115** | 0.115 |
| 5 | Bedroom dresser | vs | Bathtub | 1 | 8.84546 | 0.35602 | **0.0002** | **0.002** |
| 6 | Bedroom dresser | vs | Kitchen bench | 1 | 15.30893 | 0.48896 | **0.0001** | **0.001** |
| 7 | Bedroom dresser | vs | Hallway floor | 1 | 1.59497 | 0.09065 | 0.1247 | 1 |
| 8 | Bathtub | vs | Kitchen bench | 1 | 7.80162 | 0.32777 | **0.0001** | **0.001** |
| 9 | Bathtub | vs | Hallway floor | 1 | 7.45427 | 0.31782 | **0.0001** | **0.001** |
| 10 | Kitchen bench | vs | Hallway floor | 1 | 13.89251 | 0.46475 | **0.0001** | **0.001** |

**Table S13.** Beta diversity statistics based on Bray-Curtis dissimilarities
(9999 permutations). Bolded values indicate statistical significance*.*

| **PERMANOVA (pairwise)  Subset of Samples: Surface, Site: SEMI-URBAN, Factor: Surface Type** | | | | | | | | | |
| --- | --- | --- | --- | --- | --- | --- | --- | --- | --- |
| # | **pairs** | | | **Df** | **F. Model** | **R^2^** | ***p*-value** | ***p-*adjusted** |  |
| 1 | Bookcase shelf | vs | Bedroom dresser | 1 | 0.98586 | 0.05804 | 0.3669 | 1 |  |
| 2 | Bookcase shelf | vs | Bathtub | 1 | 6.70061 | 0.29517 | **0.0001** | **0.0015** |  |
| 3 | Bookcase shelf | vs | Kitchen bench | 1 | 10.59845 | 0.39846 | **0.0003** | **0.0045** |  |
| 4 | Bookcase shelf | vs | Hallway floor | 1 | 6.00478 | 0.27288 | **0.0001** | **0.0015** |  |
| 5 | Bookcase shelf | vs | Bedroom carpet | 1 | 19.6685 | 0.55142 | **0.0001** | **0.0015** |  |
| 6 | Bedroom dresser | vs | Bathtub | 1 | 6.43737 | 0.2869 | **0.0002** | **0.003** |  |
| 7 | Bedroom dresser | vs | Kitchen bench | 1 | 10.24975 | 0.39047 | **0.0001** | **0.0015** |  |
| 8 | Bedroom dresser | vs | Hallway floor | 1 | 6.35868 | 0.28439 | **0.0001** | **0.0015** |  |
| 9 | Bedroom dresser | vs | Bedroom carpet | 1 | 18.98847 | 0.54271 | **0.0001** | **0.0015** |  |
| 10 | Bathtub | vs | Kitchen bench | 1 | 4.07342 | 0.20293 | **0.0002** | **0.003** |  |
| 11 | Bathtub | vs | Hallway floor | 1 | 4.29407 | 0.21159 | **0.0003** | **0.0045** |  |
| 12 | Bathtub | vs | Bedroom carpet | 1 | 8.63558 | 0.35053 | **0.0001** | **0.0015** |  |
| 13 | Kitchen bench | vs | Hallway floor | 1 | 6.4386 | 0.28694 | **0.0001** | **0.0015** |  |
| 14 | Kitchen bench | vs | Bedroom carpet | 1 | 11.44211 | 0.41695 | **0.0001** | **0.0015** |  |
| 15 | Hallway floor | vs | Bedroom carpet | 1 | 2.42539 | 0.13163 | **0.0008** | **0.012** |  |
